# Serum miRNA Profile in Diabetic Patients With Ischemic Heart Disease as a Promising Non-Invasive Biomarker

**DOI:** 10.3389/fendo.2022.888948

**Published:** 2022-05-18

**Authors:** Agnieszka Bielska, Magdalena Niemira, Witold Bauer, Iwona Sidorkiewicz, Anna Szałkowska, Anna Skwarska, Justyna Raczkowska, Damian Ostrowski, Kamil Gugała, Sławomir Dobrzycki, Adam Krętowski

**Affiliations:** ^1^Clinical Research Centre, Medical University of Białystok, Białystok, Poland; ^2^Department of Leukemia, The University of Texas M.D. Anderson Cancer Center, Houston, TX, United States; ^3^Department of Invasive Cardiology, Medical University of Białystok, Białystok, Poland; ^4^Department of Endocrinology, Diabetology and Internal Medicine, Medical University of Białystok, Białystok, Poland

**Keywords:** miRNA, ischemic heart disease, diabetes, miRNA profiling, biomarker

## Abstract

The increasing morbidity and mortality of type 2 diabetic mellitus (T2DM) patients with ischemic heart disease (IHD) highlight an urgent need to identify early biomarkers, which would help to predict individual risk of development of IHD. Here, we postulate that circulating serum-derived micro RNAs (miRNAs) may serve as potential biomarkers for early IHD diagnosis and support the identification of diabetic individuals with a predisposition to undergo IHD. We obtained serum samples from T2DM patients either with IHD or IHD-free and analysed the expression levels of 798 miRNAs using the NanoString nCounter technology platform. The prediction of the putative miRNAs targets was performed using the Ingenuity Pathway Analysis (IPA) software. Gene Ontology (GO) analysis was used to identify the biological function and signalling pathways associated with miRNA target genes. Hub genes of protein-protein interaction (PPI) network were identified by STRING database and Cytotoscape tool. Receiver operating characteristic (ROC) analysis was used to assess the diagnostic value of identified miRNAs. Real-time quantitative polymerase chain reaction (qRT-PCR) was used for nCounter platform data validation. Our data showed that six miRNAs (miR-615-3p, miR-3147, miR-1224-5p, miR-5196-3p, miR-6732-3p, and miR-548b-3p) were significantly upregulated in T2DM IHD patients compared to T2DM patients without IHD. Further analysis indicated that 489 putative target genes mainly affected the endothelin-1 signalling pathway, glucocorticoid biosynthesis, and apelin cardiomyocyte signalling pathway. All tested miRNAs showed high diagnostic value (AUC = 0.779 - 0.877). Taken together, our research suggests that circulating miRNAs might have a crucial role in the development of IHD in diabetic patients and may be used as a potential biomarker for early diagnosis.

## Introduction

Diabetes mellitus is a group of endocrine and metabolic disorders characterised by insufficient insulin secretion to maintain the right blood glucose level. According to the International Diabetes Federation, 463 million people had diabetes in 2019, and this number is expected to rise in the following year’s (1). Several forms of diabetes are distinguished; however, type 2 diabetes mellitus (T2DM) is the most common and accounts for over 90% of cases (2). T2DM is characterised by cell resistance to the normal concentration of insulin circulating in the blood. With the progression of the disease, pancreatic β-cell may also become dysfunctional and inevitably stop producing insulin (3). The most common long-term complications of diabetes is ischemic heart disease (IHD) (4, 5), and it is well known as the leading cause of morbidity and mortality in diabetes (6). IHD results from an imbalance between blood supply and oxygen demand in myocardial cells caused by different degrees of coronary artery obstruction, and occurs when atherosclerosis develops in the coronary artery.

Ischemia is related to inadequate oxygen supply and reduced availability of nutrients, and incomplete removal of metabolic products (7). Patients with T2DM have a higher risk of developing IHD and mortality following IHD than healthy ones (8).

Diabetes complications, such as heart diseases, develop much earlier before diagnosing (9). To prevent the progression of the disease, special attention should be paid to its early detection. Despite the rapid progress in cardiovascular research, there is no reliable tool for prompt diagnosis and identification of people at risk of developing IHD. Coronary angiography remains the gold standard in the diagnosis of IHD. Unfortunately, it is an invasive medical procedure that uses contrast dye to detect blockages in coronary arteries at x-ray pictures (10). Additionally, this method can only diagnose the disease at a later stage. Therefore, early and noninvasive detection of this condition at the initial state or diagnosis of diabetic patients with a predisposition to IHD is crucial.

Recent research shows that inflammatory mediators like chemokines CXCL12 and macrophage migration-inhibitory factor (MIF) play an essential role in the pathology of IHD. In humans, MIF is abundantly produced by various cells in different stages of plaque development (11, 12). Elevated levels of MIF in plasma can serve as an early biomarker for acute myocardial ischemia and can be risk factor for future coronary events in IHD patients with T2DM (13, 14). It has been shown that CXCL12 levels in plasma are better predictors of IHD outcomes than traditional risk factors (15, 16). Unfortunately, the serum concentration of MIF and CXCL12 seems to be characteristic not only for ischemic heart disease but also for different inflammatory states raising the need for more selective markers for IHD diagnosis (17).

A promising tool for understanding the pathogenesis of IHD is miRNAs (18). MiRNAs are small (17-25 nucleotides) non-coding RNAs that play an essential role in regulating gene expression. MiRNAs control the expression of target genes by base pairing to the 3’ untranslated regions (3’ UTRs) of mRNA and inducing repression of the target mRNA. This effect can occur by transcript destabilisation or inhibition of translation (19). Bioinformatics predictions indicate that one miRNA could target more than a hundred mRNAs (20). MiRNAs participate in critical biological processes such as proliferation, differentiation, and apoptosis of cells (21). Abnormal expression of miRNAs is associated with various diseases, such as cancer, cardiovascular diseases or metabolic disorders. It is commonly known that levels of specific circulating miRNAs might be predictive for long-term diabetes complications (22). MiRNAs may be a helpful biomarker because of their stability in biofluids even after prolonged collection and several freezing-thaw cycles (23). Moreover, those small particles can be easily collected and measured with specific, sensitive assays (22).

MiRNAs such as miR-92a, miR-503, and miR-126 might control and regulate angiogenesis, crucial for the repair of myocardial cells after ischemic injury (24, 25). MiR-155 and miR-342-5p are involved in vascular inflammation by modulation of inflammatory response and atherosclerosis progression (26). MiR-125b and miR-205 can regulate vascular calcification that contributes to atherosclerosis (27, 28). Decreased level of miR-126 is known as a predictor of diabetes, and it also occurs in patients with IHD (21). In diabetes, there is an established connection between miR-223 and activation of platelets, the latter of which significantly contributes to the development of cardiac disease (29). However, despite evident progress, our understanding of the regulation and function of specific miRNAs in IHD is still limited.

In the present study we investigated the differential expression of IHD-associated miRNAs in the serum samples from T2DM patients with and without IHD using the nCounter platform, a novel technique offering a high level of precision and sensitivity without amplification reaction (30).

## Materials and Methods

### Baseline Characteristics of the Patients

To diagnose IHD in over 600 patients who participated in the cohort study conducted in the Department of Invasive Cardiology, Medical University of Bialystok, coronary angiography was performed. This procedure has distinguished two subsets of T2DM patients – with IHD (*n* = 24) and without IHD *(n* = 20), IHD group (*n* = 9) and the control group without diabetes or prediabetes and IHD (*n* = 16). T2DM was confirmed according to the Diabetes Poland criteria (31). Exclusion criteria for this study included: type 1 diabetes mellitus, latent autoimmune diabetes of adults, other T2DM complications (retinopathy, neuropathy, nephropathy, peripheral artery disease, stroke, cerebrovascular disease), previous myocardial infraction, percutaneous coronary intervention, other inflammatory states (rheumatoid arthritis, systemic sclerosis), cancers, human immunodeficiency virus (HIV) or hepatitis C virus (HCV) infection, recent surgery, alcohol consumption, and smoking. A group of 69 individuals was qualified for further analysis. Serum samples were collected, centrifuged and stored at −80°C. This study was approved by the Bioethics Committee of the Medical University of Bialystok, Poland (approval numbers: R-I-002/583/2019 and APK.002.35.2021) and was performed according to the principles of the Declaration of Helsinki.

### MiRNAs Isolation

The miRNeasy Serum/Plasma Advanced Kit (Qiagen, Germany) was used for RNA extraction (smaller than 1000 nucleotides) using 200 μL of serum aliquots from one patient according to the manufacturer’s instructions. The miRNA concentration was measured by The Qubit microRNA Assay Kit (Invitrogen, California, CA, United States) with the Qubit 3.0 Fluorometer.

### Detection of miRNAs Profile

A total of 69 samples were prepared for nCounter miRNA expression profiling according to the manufacturer’s recommendations (NanoString Technologies, USA). A three ng of isolated microRNA were used as input material. Unique DNA tags were ligated onto the 3′ end of each mature miRNA, providing an identifier for each miRNA in the sample. Tagging was performed in the ligation reaction followed by an overnight hybridization (65°C) to nCounter Reporter and Capture probes that allowed complex sequence-specific probes with targets. After hybridization, samples were placed into the nCounter Prep Station for automated sample purification and target/probe complexes immobilization on the cartridge for data collection. Each sample was scanned for 555 FOV (fields of view) on the nCounter Digital Analyzer (NanoString Technologies, USA) to count individual fluorescent barcodes and quantify target RNA molecules present in each sample. NanoString raw data were analysed with nSolver software (NanoString Technologies, USA).

### Measurement of MIF and CXCL12 in Serum

The concentration of MIF and CXCL12 in serum was determined in duplicate samples by enzyme-linked immunosorbent assay (ELISA) (Quantikine Human M-CSF Immunoassay; R&D systems, Abingdon, United Kingdom), according to the manufacturer’s recommendations.

### Validation of the NanoString Results by Real-Time Quantitative Polymerase Chain Reaction (qRT-PCR)

The serum of 22 T2DM and 26 T2DM IHD miRNA samples were reverse-transcribed using a miRCURY LNA RT Kit (Qiagen, Hilden, Germany), according to the manufacturer’s instructions, on a Proflex thermal cycler (Thermo Fisher Scientific, Waltham, USA). Subsequently, the qRT-PCR reaction was performed using specific primers and a miRCURY LNA SYBR Green PCR Kit (Qiagen, Germany). The samples were run on the qPCR plates in duplicate on the LightCycler 480 Real-Time PCR System (Roche, Switzerland). Based on the NormFinder (32), miR-103a-3p and miR-199b-5p were used as endogenous reference miRNAs. The primers efficiency for targets and reference miRNAs has been checked. After calculating the qPCR efficiency, relative expression levels of miRNAs were calculated using the delta-delta *Ct* (2^–ΔΔCt^) method. *Ct* is the threshold cycle which is a point when fluorescence reading surpasses a set baseline. This method calculates samples’ relative fold expression, using a reference miRNA as the normalizer (33).

### Statistical Analysis

Based on similar previous experiments and pilot data on NanoString nCounter miRNA Expression Assay we have calculated the minimal number of samples per experimental group (T2DM IHD or T2DM) in order to detect 1.5 fold differences in relative miRNA expression level between groups at the true positives detection powers of 80% and 90% under *p*=0.05 (34). We have used RNASeqPower *R* package (35) applying the statistics data covering obtained counts and coefficients of variations per different groups. We have estimated that to obtain 80% power we would need 12 samples, whereas to obtain high power of 90% we would need 16 samples per group. Finally our groups consisted of 20 samples in T2DM and 24 in IHD T2DM group thus allowing for more than 90% power under *p*=0.05. Statistical analyses were performed using STATISTICA version 13.1 (StatSoft, Tulsa, Oklahoma). Preliminary statistical analysis (Shapiro–Wilk test) revealed that the studied parameters did not follow a normal distribution. The ANOVA Kruskal-Wallis test followed by Dunn’s test was performed to examine the difference in clinical parameters between the groups. miRNAs were tested for differential expression using nSolver 4.0 Analysis software (NanoString), including normalization using the positive ligation controls. The threshold count value was set to 20. The *p*-value was adjusted using the False Discovery Rate (FDR) correction for multiple comparisons limited to 0.05. Ingenuity Pathway Analysis (QIAGEN Inc.) was performed to generate a list of predicted targeted genes for studied miRNAs and identify canonical pathways. To find the highly connected hub genes of six miRNAs in the protein-protein network (PPI) STRING database (http://string-db.org) (36), Cytoscape version 3.9.0 (http://cytoscape.org/) (37) and its plugin (cytoHubba) were applied. GO and functional annotation clustering were carried out using the KEGG (Kyoto Encyclopedia of Genes and Genomes) Enrichment Analysis; g:Profiler (https://biit.cs.ut.ee/gprofiler/gos), and Metascape (https://metascape.org) online databases. GO analysis permits linking a list of genes to specific functional annotations categorized into functional groups (38). The Revigo web-based tool (http://revigo.irb.hr/) was used to summarise and visualise lists of Gene Ontology. The Spearman rank-order correlation coefficient (*r*) was determined to estimate the correlation between the identified miRNAs and clinical parameters. It was assumed that *r* > 0.8 indicates a strong correlation, and *r* > 0.3 shows a moderate correlation. The statistical significance level was set at *p* < 0.05. Receiver operating characteristic (ROC) analysis was used to assess the diagnostic value of miRNAs, and for each miRNA, the area under the curve (AUC) was calculated. The logistic regression model was created using Weka software to assess the diagnostic values of multi-miRNAs assays.* *MiRNAs for analysis were selected using attribute selection (evaluator: Info Gain, search: Ranker). MiRNAs with the highest ranks were used for further modelling. Logistic regression, random tree, J48 tree, and naive bayes classification algorithms with 10-fold cross-validation were used to select the combination of miRNAs with the highest diagnostic value.

## Results

### Patient Baseline Characteristic

The study groups consisted of 44 patients with T2DM; 24 of them were diagnosed with IHD. Nine patients suffered only from IHD, without T2DM. Sixteen patients were control group with normal glucose tolerance and negative coronarography results. IHD and IHD-free groups did not show significant differences in clinical parameters such as duration of diabetes, platelets, fibrinogen, body mass index (BMI), glucose level, glycated haemoglobin (HbA1c) level, blood pressure, cholesterol, triglycerides (TG), low-density lipoprotein (LDL), and high-density lipoprotein (HDL). The clinical characteristics of patients enrolled in this study are summarized in **Table 1**. Patients in both diabetes groups had been treated with standard regimens and took oral hypoglycaemic medications. Patients with T2DM had been treated with derivatives of biguanide (42% of T2DM and 50% of T2DM IHD group, *p* > 0.05) and, sulfonylurea derivatives (58% of T2DM and 68% of T2DM IHD group, *p* > 0.05).

**Table 1:** Patient baseline characteristics.

Characteristics	Patients (*n* = 16) controls	Patients (*n* = 9) IHD	Patients (*n* = 20) T2DM	Patients (*n* = 24) T2DM IHD
** **	median (Q1-Q3)	median (Q1-Q3)	median (Q1-Q3)	median (Q1-Q3)
Age [years]	52.32 (49.88 - 55.91)	54.85 (50.57 - 61.20)	57.50 (52.33 - 65.17)	58.33 (53.4 - 64.58)
T2DM duration [years]	–	–	7.00 (4.00 -10.00)	5.00 (2.00-10.00)
BMI [kg/m^2^]	24.62 (22.19 - 25.66)	27.73 (26.47 - 30.39)^a*^	32.19 (28.63 - 36.41)^a****^	31.24 (27.68 - 33.20)^a***^
Platelets [10^3^/μL]	227.50 (163.00 - 268.00)	221.00 (195.00 - 248.00)	222.00 (197.00 - 243.00)	225.00 (186.00 - 322.00)
Fibrinogen [mg/dL]	333.50 (272.00 - 352.00)	362.00 (296.00 - 413.00)	352.00 (315.00 - 381.00)	416.00 (354.00 - 482.00)^a**^
Leukocytosis [10^3^/μL]	6.45 (5.49 - 7.30)	7.51 (5.60 - 8.90)	7.16 (5.40 -8.47)	7.32 (6.38 - 9.30)
Fasting glucose [mg/dL]	89.00 (85.00 - 91.00)	90.00 (84.00 - 100.00)	122.00 (118.00 - 165.00)^a****^,b^***^	129.00 (104.00 - 176.00)^a***^,b^**^
HbA1c [%]	5.50 (5.50 - 5.65)	5.50 (5.30 - 5.70)	6.20 (5.70 - 7.50)^a*^,b^*^	7.60 (6.20 - 8.80)^a***^,b^***^
Systolic blood pressure [mmHg]	120.00 (115.00 - 130)	120.00 (110.00 - 140.00)	140.00 (120.00 – 155.00)^a*^	140.00 (130.00 – 155.00)^a***^,b^*^
Diastolic blood pressure [mmHg]	80.00 (70.00 - 80.00)	80.00 (80.00 - 100.00)	80.00 (80.00 - 90.00)	85.00 (80.00 - 90.00)
Cholesterol [mg/dL]	194.00 (162.00 - 209.00)	176.00 (163.00 - 196.00)	198.50 (172.00 - 224.00)	179.50 (138.00 - 211.00)
TG [mg/dL]	109.00 (86.00 - 153.00)	134.00 (109.00 - 183.00)	124.00 (90.00 - 202.00)	179.00 (109.00 - 206.00)
LDL [mg/dL]	112.00 (92.00 - 132.00)	106.00 (102.00 - 117.00)	118.50 (102.00 - 135.00)	102.00 (66.00 - 133.00)
HDL [mg/dL]	49.00 (40.00-62.00)	35.00 (33.00 - 45.00)	46.00 (41.00 - 57.00)^b*^	39.00 (34.00 - 50.00)

a Significantly different from control group;^b^Significantly different from IHD group *p<0.05; **p<0.01; ***p<0.001; ****p<0.0001; CI, confidence interval; the p-value describes the significance of the difference between patients with T2DM without IHD compared to patients with T2DM and IHD using ANOVA Kruskal-Wallis test followed by Dunn’s test.

### Baseline Levels of Circulating miRNAs

To determine the differentially expressed miRNAs (DEMs) in serum of diabetic patients with IHD (*n* = 24) compared to patients with T2DM (*n* = 20), patients with IHD (*n* = 9), and control group (*n* = 16), we performed the expression analysis of 798 unique miRNAs using the nCounter platform. nSolver 4.0 analysis indicated that 14 miRNAs were identified as differentially expressed between T2DM IHD and T2DM groups. All of them were upregulated in serum samples of T2DM patients with IHD when compared to diabetic patients without IHD (|FC| ≥ 1.5, FDR ≤ 0.05) (**Supplementary Table 1**). Altogether 155 DEMs were found between the T2DM IHD compared to the control group (**Supplementary Table 2**). Additionally, 155 DEMs were found between T2DM and control group (**Supplementary Table 3**). Furthermore, 221 DEMs were found between T2DM IHD and IHD groups (**Supplementary Table 4**). Those comparisons allowed extract six DEMs typical only for T2DM IHD patients (**Table 2**).

**Table 2:** MiRNAs upregulated in T2DM IHD compared with T2DM patients.

miRNA	FC	*p*-value	FDR
miR-615-3p	2.45	0.00000497	0.00
miR-3147	2.35	0.00000200	0.00
miR-1224-5p	1.68	0.00001305	0.00
miR-5196-3p + miR-6732-3p	1.56	0.00040774	0.01
miR-548b-3p	1.55	0.0002042	0.01

FC, fold change; FDR, false discovery rate, adjusted p-value; FC ≥ 1.5; FDR ≤ 0.05.

### Functional Enrichment Analysis in T2DM IHD Patient Samples

**Figure 1:**
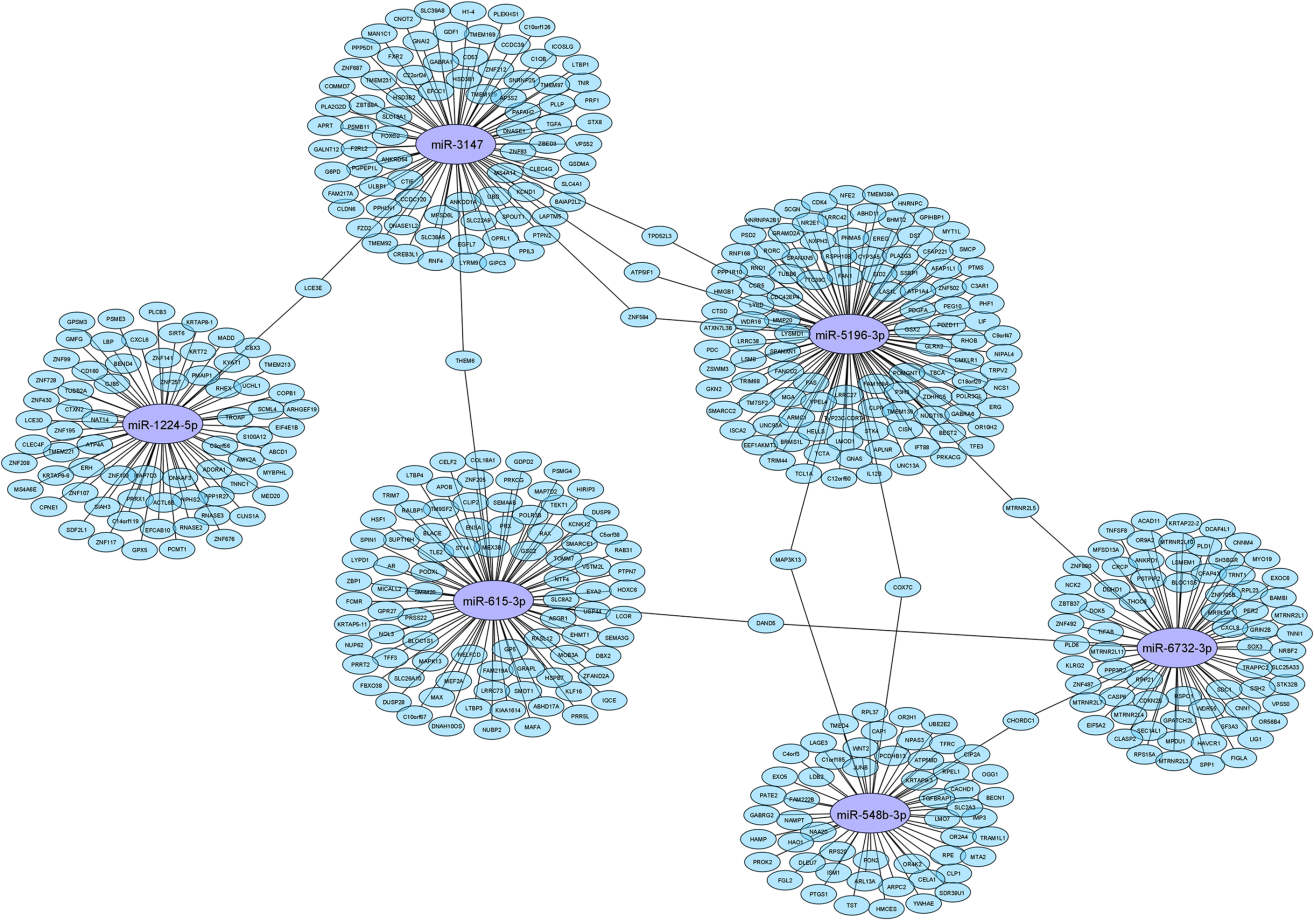
Network of connection between tested miRNAs and their target genes.

The IPA analysis demonstrated 489 putative target genes for six DE miRNAs (**Supplementary Table 5**). To visualise connections between tested miRNAs and target genes, Cytoscape version 3.9.0 (http://cytoscape.org/) was used. The obtained network of connections is shown in **Figure 1**.

**Figure 2:**
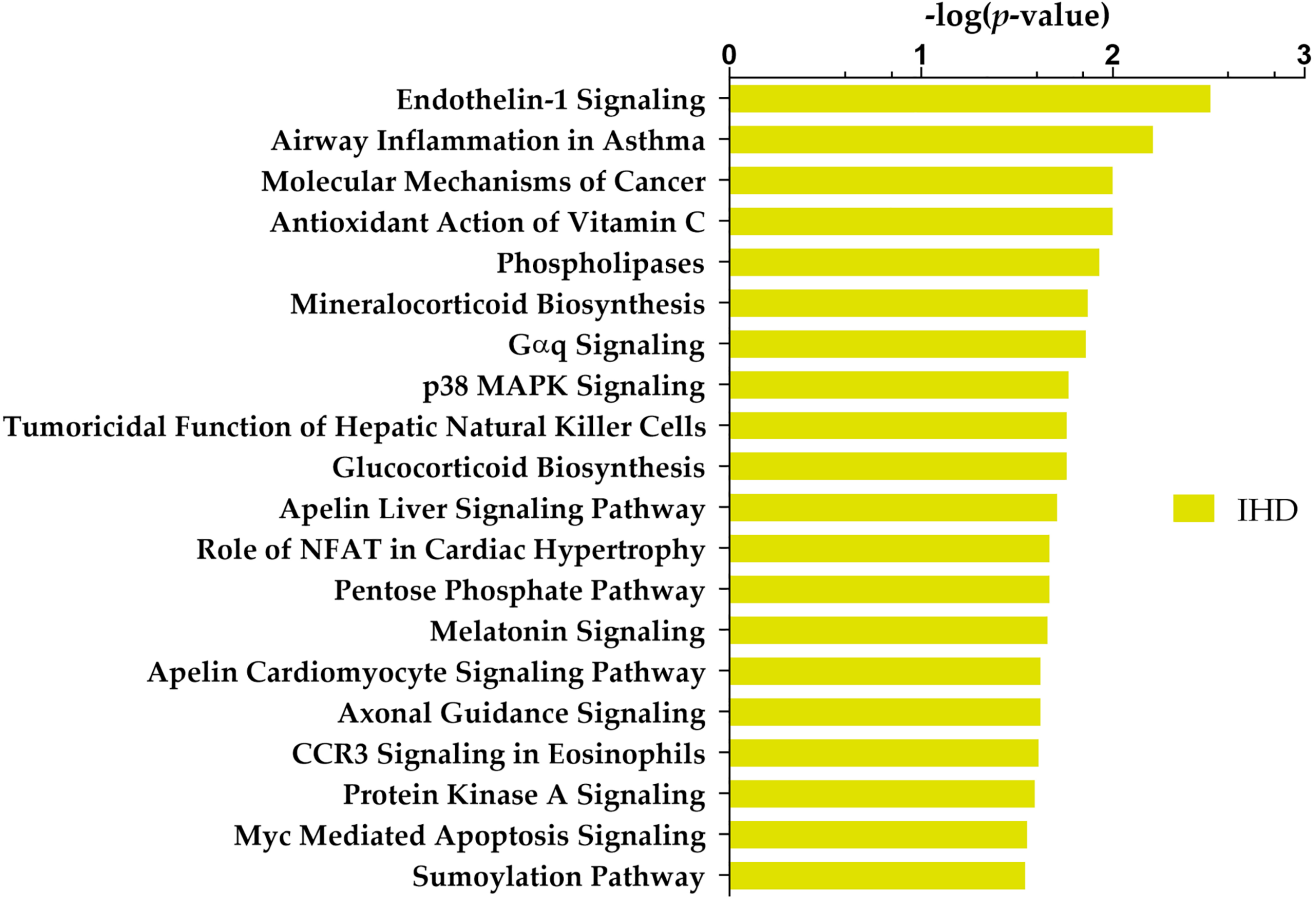
Top 20 Ingenuity Pathway Analysis (IPA) canonical pathways most significantly changed in T2DM patients with IHD compared to individuals without IHD. The x-axis shows the -log of *p*-value calculated by the Benjamini-Hochberg (B-H) method.

Ingenuity core analysis identified 36 altered canonical pathways, including the endothelin-1 signalling pathway, glucocorticoid biosynthesis, and apelin cardiomyocyte signalling pathway (**Supplementary Table 6**). The top 20 deregulated canonical pathways are shown in **Figure 2**.

**Figure 3:**
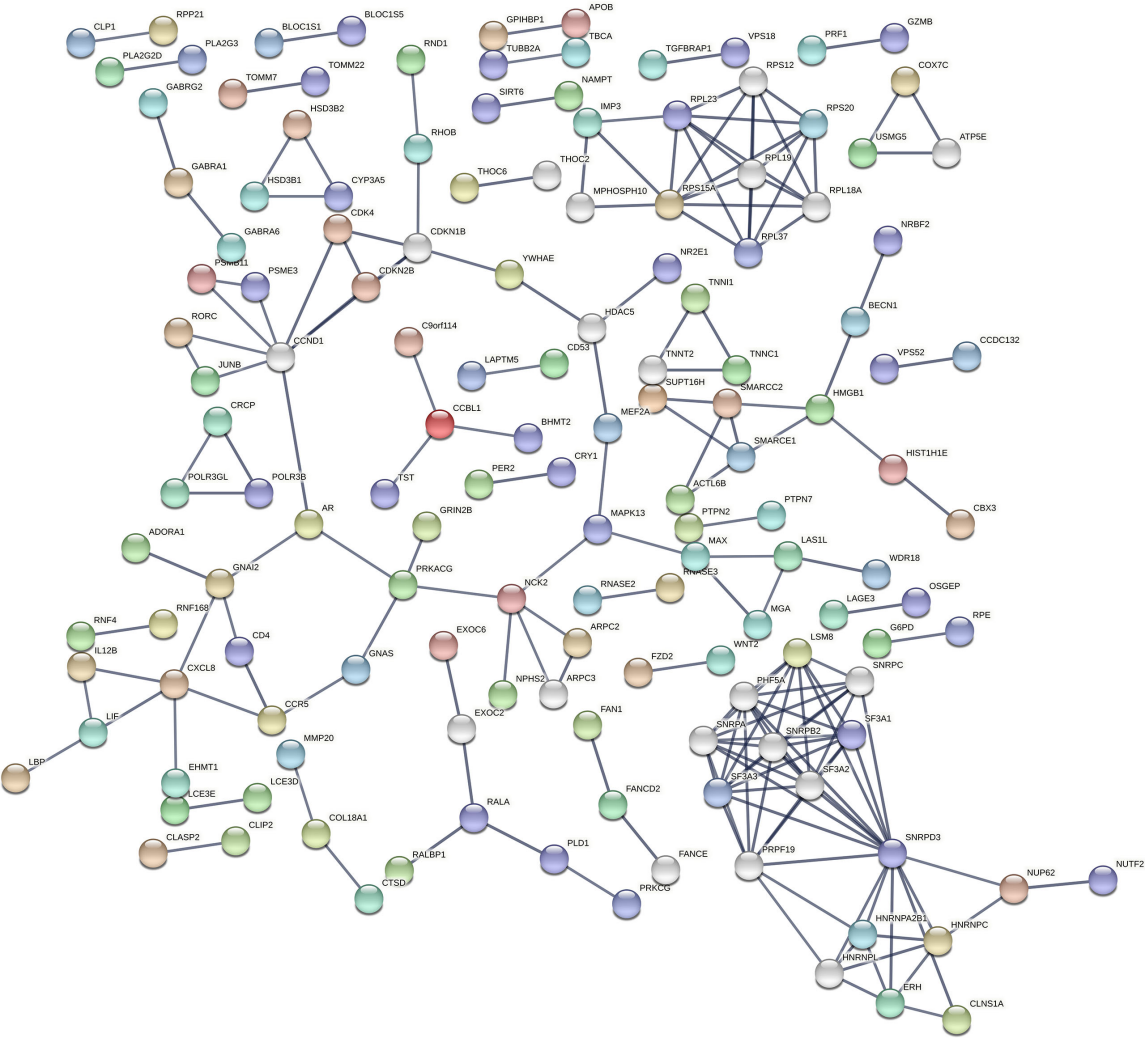
Protein-protein interaction (PPI) network of DE miRNA’s targeted genes by STRING. STRING is a database of known and predicted protein-protein interactions, including physical and functional associations based on text mining of the scientific literature, other databases, computational predictions, and knowledge transfer between organisms. The network gives information on the degree of overall connectivity across imputed genes. The interaction score was set to the highest confidence (0.9), showing a high probability of the association being true.

STRING database (36) (https://string-db.org/) in Cytoscape tool (37) and the *cytoHubba* plugin (39) were used to visualise the protein-protein interaction (PPI) network of the DE miRNA target genes and identify top 10 hub genes (**Figure 3**)

**Figure 4:**
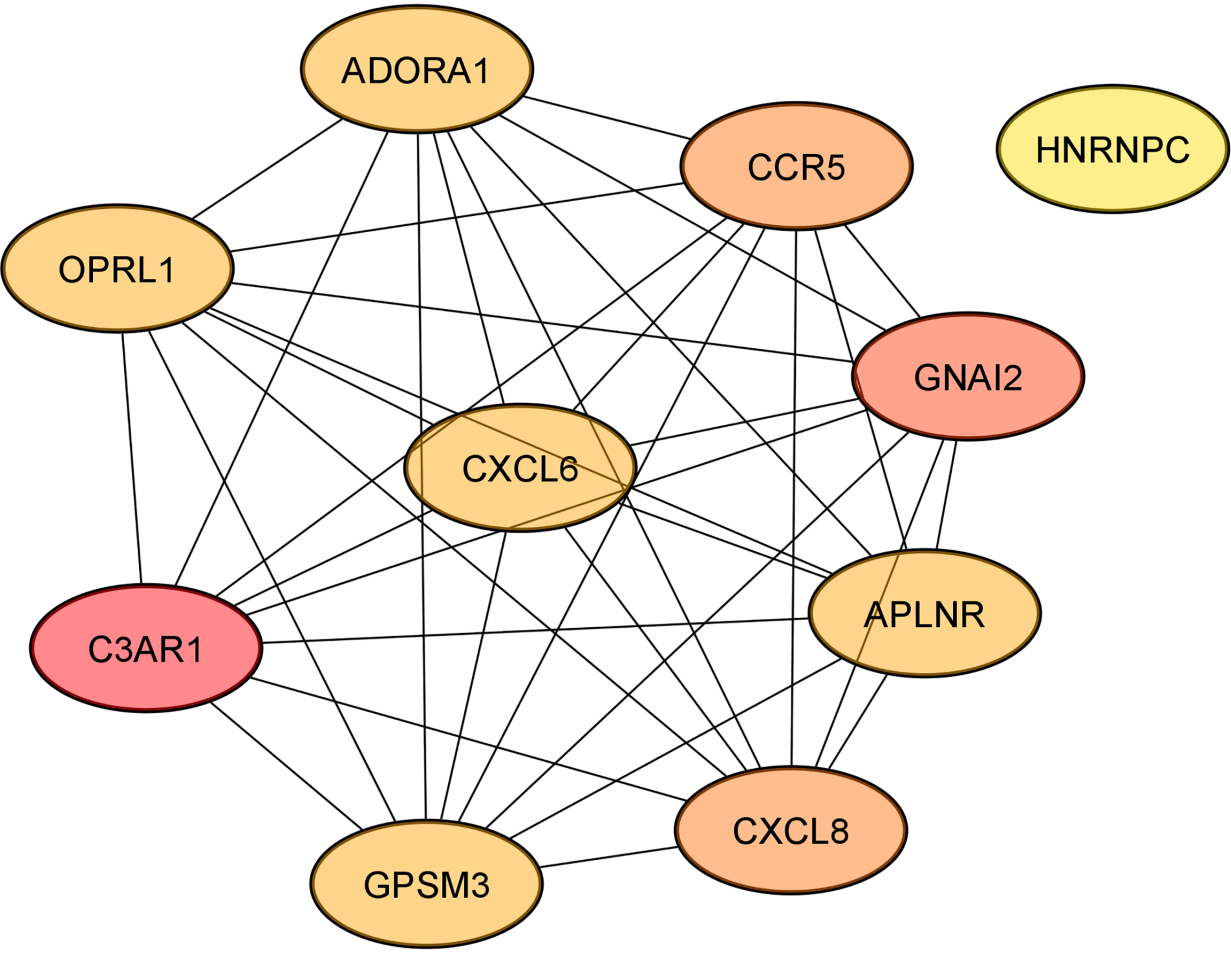
The networks of the top 10 hub genes.

According to cyto*Hubba* plugin’s MCC (The Maximal Clique Centrality) ranking, the top 10 hub genes in the PPI network were *C3AR1* (Complement C3a Receptor 1), *CCR5* (C-C Motif Chemokine Receptor 5), *APLNR* (Apelin Receptor), and *HNRNPC* (Heterogeneous Nuclear Ribonucleoprotein C) targeted by miR-5196-3p; *GNAI2* (G Protein Subunit Alpha I2) and *OPRL1* (Opioid Related Nociceptin Receptor 1) targeted by miR-3147; *CXCL8* (C-X-C Motif Chemokine Ligand 8) targeted by miR-6732-3p, *ADORA1* (Adenosine A1 Receptor), *CXCL6* (C-X-C Motif Chemokine Ligand 6), and *GPSM3* (G Protein Signaling Modulator 3) targeted by miR-1224-5p (**Figure 4**).

**Figure 5:**
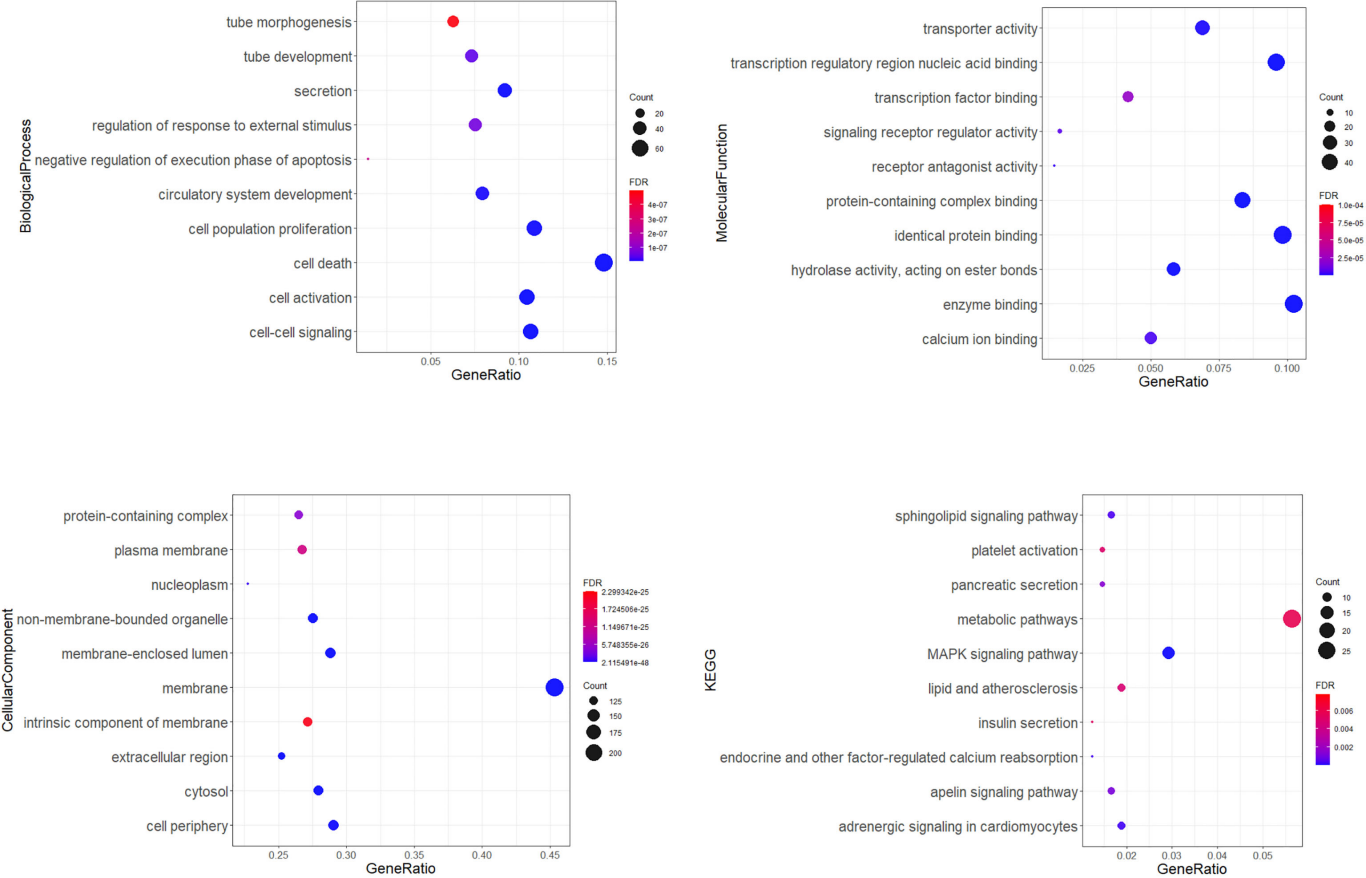
Gene ontology (GO) enrichment analysis. Dot plots showing top 10 significantly enriched GO terms of the target genes in the biological process, molecular function, cellular components, and top 10 KEGG (Kyoto Encyclopedia of Genes and Genomes) connected with cardiovascular diseases and diabetes. The colors of the nodes are illustrated from red to blue in descending order of the FDR adjusted *p*-value. The sizes of the nodes are shown from small to large in ascending order of gene counts. The x-axis represents the gene ratio, and the y axis represents the GO or KEGG terms. GO: Gene Ontology; KEGG: Kyoto Encyclopedia of Genes and Genomes.

Gene ontology (GO) enrichment analysis identified biological pathways and processes associated with the target genes. The GO-biological process (BP) analysis identified the most significant pathways included cell death, cell activation, cell-cell signalling, secretion, cell population proliferation, circulatory system development, tube development, regulation of response to external stimulus, negative regulation of execution phase of apoptosis, and tube morphogenesis. Transcription regulatory region nucleic acid binding, protein-containing complex binding, enzyme binding, hydrolase activity, acting on ester bonds, identical protein binding, transporter activity, receptor antagonist activity, calcium ion binding, signalling receptor regulator activity, and transcription factor binding were pointed as the main important in GO-molecular function analysis. According to the GO-cellular component analysis, target genes were mainly enriched in the membrane, non-membrane-bounded organelle, membrane-enclosed lumen, cytosol, extracellular region, cell periphery, nucleoplasm protein-containing complex, plasma membrane, and an intrinsic component of membrane. KEGG pathway enrichment analysis showed pathways involved in cardiovascular diseases and diabetes development (**Figure 5**).

### Levels of MIF and CXCL12 in Serum

No significant differences were observed between the serum concentration of MIF and CXCL12 in T2DM IHD and T2DM groups (*p*>0.05). The median values of MIF were 0.65 ng/ml in the T2DM IHD group and 0.85 ng/ml in the T2DM group. The median values of CXCL12 in the T2DM IHD and T2DM groups were 2001.54 pg/ml and 2158.37 pg/ml, respectively (*p* > 0.05). Both levels of CXCL12 were significantly different in the T2DM and T2DM IHD compared to the control group (**Supplementary Table 7**). These results indicate that neither MIF nor CXCL12 can serve as good IHD prognostic markers in T2DM patients.

### Correlation of Circulating miRNAs With Clinical Data

**Figure 6:**
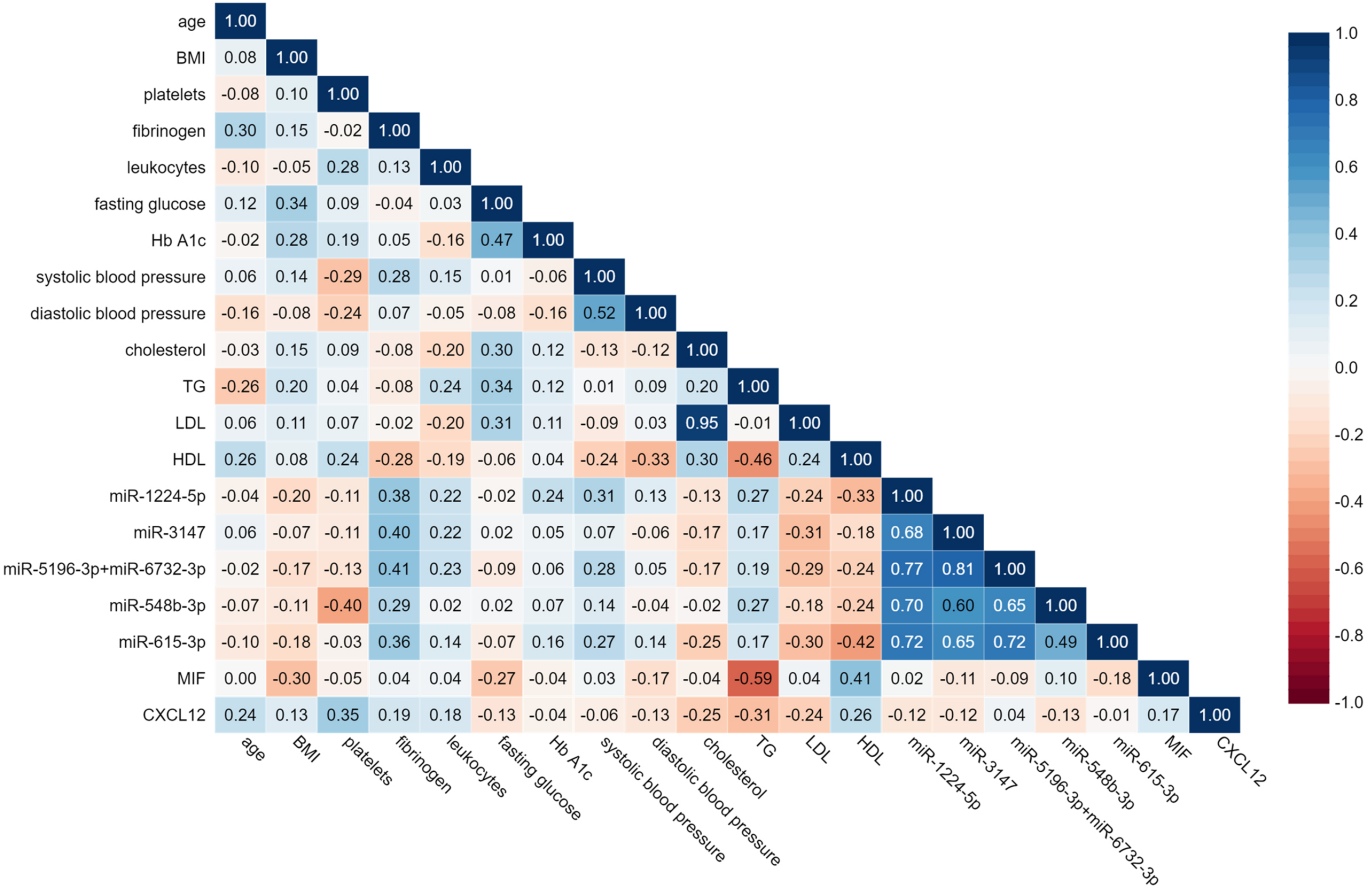
Spearman’s rank-matrix correlation between tested miRNAs, MIF, CLXC12 and clinical parameters. The blue colour indicates a positive correlation, and red indicates the opposite.

A Spearman’s rank-order regression analysis was performed to assess the relationship between miRNAs levels and clinical parameters of patients (**Figure 6**). The analysis indicated significant moderate correlation between fibrinogen and miR-1224-5p, miR-3147, miR-5196-3p+miR-6732-3p, and miR-615-3p (*r* = 0.38; *r* = 0.40; *r* = 0.41; *r* = 0.36 respectively; *p* < 0.05). Platelet count was negatively correlated with miR-548b-3p (*r* = -0.4; *p* < 0.05). Systolic blood pressure was correlated with miR-1224 (*r* = 0.31; *p* < 0.05). Analysis indicated moderate correlation between HDL and miR-615-3p (*r* = -0.42, *p* < 0.05). Statistical analysis showed strong positive correlation between serum levels of all studied miRNAs. The strongest positive correlation was indicated between miR-5196-3p+miR-6732-3p and miR-3147 (*r* = 0.81; *p* < 0.001). MiR-3147 was also correlated with miR-615 and with miR-548b-3p (*r* = 0.65; *r* = 0.60 respectively; *p* < 0.001). MiR-1224-5p showed correlation with miR-548b-3p, miR-5196-3p+miR-6732-3p, miR-3147 (*r* = 0.70; *r* = 0.77; *r* = 0.68; *p* < 0.001). MiR-615-3p showed correlation with miR-5196-3p+miR-6732-3p and miR-548b-3p (*r* = 0.72; *r* = 0.49 respectively; *p* < 0.001). No significant correlations between MIF and CXCL12 levels and upregulated miRNAs were found.

### Evaluation of Diagnostic Values of Tested miRNAs

**Figure 7:**
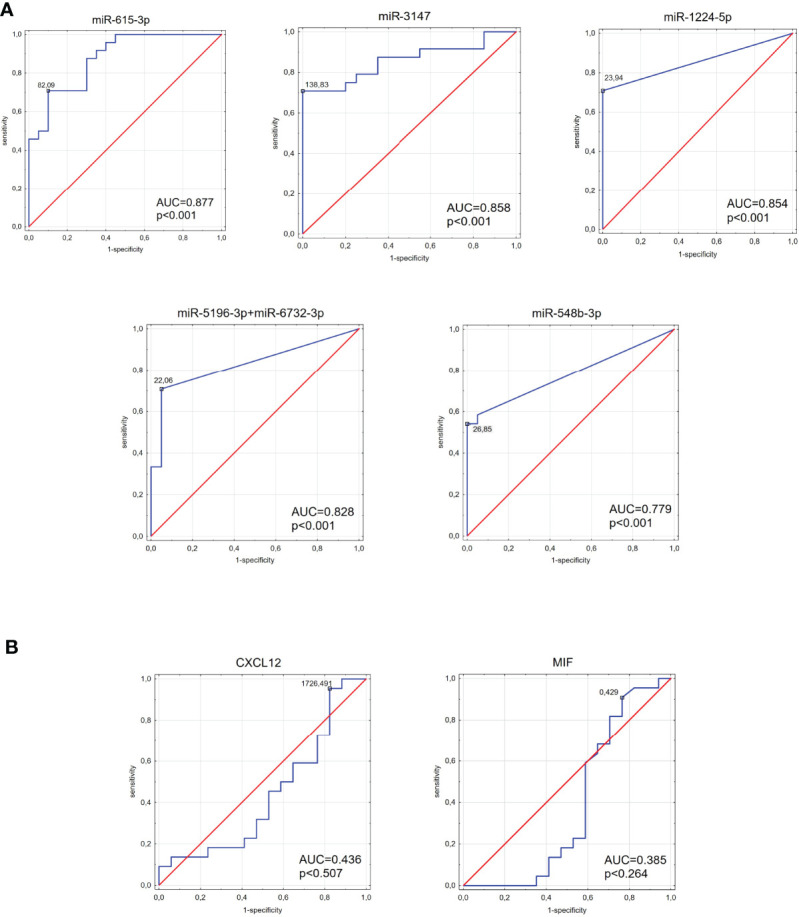
The area under the curve (AUC) of the receiver operating characteristic (ROC) curves for tested miRNAs **(A)** and MIF and CXCL12 proteins **(B)**. ROC analysis was carried out to evaluate the diagnostic potential of selected miRNA as a predictive biomarker of type 2 diabetes compared to MIF and CXCL12 proteins.

ROC curve analysis was performed to estimate the possible roles of identified miRNAs as biomarkers for IHD in diabetic patients (**Figure 7**). AUC (area under the ROC curve) for all six miRNAs reached significance comparing to AUC = 0.500 (*p* < 0.001 in all cases). The AUC values > 0.800 were found for miR-1224-5p, miR-3147, miR-5196-3p+miR-6732-3p and miR-615-3p. AUC score for the miR-548b-3p was also high (AUC = 0.779). The AUC values for MIF and CXCL12 were less than 0.500 (AUC = 0.5 borderline of the diagnostic usefulness of the test).

### Logistic Regression Model

To investigate the possible increase of diagnostic value by simultaneous consideration of multiple DE miRNAs, logistic regression models were developed using Weka 3.8.6 (The University of Waikato, Hamilton, New Zealand) software (40). The parameters of the models and the standard quality measures are summarized in the **Table 3**. The highest AUC was obtained for a combination of miR-3147 and miR-615-3p (AUC = 0.935). This model had a higher diagnostic value compared to the highest AUC for miRNA used separately.

**Table 3:** Summary of the basic parameters and common quality measures of the models.

model	TP rate	FP rate	Precision	AUC	Intercept	Coefficients
x1 = miR-3147	0.864	0.147	0.866	0.935	28.56	a1 = -9.03
x2 = miR-615-3p						a2 = -6.87
x1 = miR-548b-3p	0.841	0.166	0.841	0.929	20.30	a1= -7.06
x2 = miR-615-3p						a2= -7.22
x1 = miR-3147	0.886	0.120	0.887	0.927	26.78	a1 = -6.49
x2 = 548b-3p						a2 = -3.09
x3 = 615-3p						a3 = -6.54
x1 = 1224-5p	0.795	0.204	0.797	0.906	20.68	a1 = -8.78
x2 = 615-3p						a2 = -5.87
x1 = miR-3147	0.795	0.212	0.795	0.881	28.00	a1 = -6.69
x2 = miR-615-3p						a2 = -6.47
x3 = 1224-5p						a3 = -2.06
x4 = 548b-3p						a4 = -2.27
x5 = 5196-3p+miR-6732-3p						a5 = 0.55

TP, true positive; FP, false positive; AUC, area under the curve of the receiver operating characteristic.

### Data Validation

**Figure 8:**
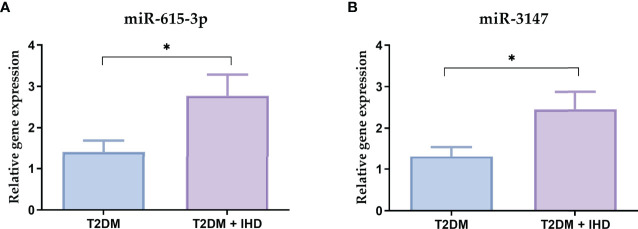
Relative mRNA expressions of the miR-615-3p **(A)** and miR-3147 **(B)** in the serum samples in the group of 22 T2DM and 26 T2DM IHD patients. Each bar represents the mean ratio of gene expression and miR-103a-3p and miR-199b-5p ± standard error of the mean. Significance: U Mann-Whitney test; *p < 0.05 in comparison to control.

To verify the results of the NanoString analysis, the most upregulated miRNAs from the miRNA profiling (miR-615-3p, miR-3147) were validated in the group of 22 T2DM and 26 T2DM IHD patients. In comparison with the results from the nCounter platform to qRT-PCR validation showed similarity between the expression patterns of tested miRNA (**Figure 8**).

## Discussion

T2DM is a complex metabolic disorder involving multiple genes that affect different signalling pathways. Diabetes is a known risk factor for IHD, and patients with higher HbA1c levels are more at risk of cardiovascular mortality (41). The development of new molecular biomarkers of identification T2DM patients with risk of IHD can improve the care of such patients. MiRNAs, as essential mediators of cell-to-cell communication, have critical roles in the epigenetic regulation of metabolic, inflammatory, and antiangiogenic pathways in diabetes-related to long term complications (42). The latest research show that miRNA can play an important role as a biomarker for diabetes and its complications (3). The potential for biofluids–derived miRNA to serve as a diagnostic tool has stimulated a wide range of studies regarding the disease-specific expression of miRNA and its stability (43). To find the unique miRNA profile in T2DM patients with IHD, we used a state-of-the-art NanoString nCounter platform that provides the opportunity to profile a large number of miRNAs that have not been previously investigated about IHD in T2DM.

In comparison to other methods of miRNA detection, the NanoString offers maximal sensitivity and specificity with high-quality data due to the elimination of amplification (44). It has been shown that this platform detects miRNAs in biofluids with sensitivity and specificity more significant than other miRNA detection methods like qPCR or microarrays (45). Moreover, this technique represents a more accessible and accurate method for everyday clinical practice because of the partially automated process that eliminates the risk of errors (46). To our best knowledge, this study is the first miRNA profiling in the serum of IHD patients using the nCounter platform, which seems to be an optimal strategy to identify novel biomarker candidates for IHD prognosis.

Our research revealed six DE miRNAs in patients with T2DM and IHD compared to diabetic individuals without IHD. No significant expression of those miRNAs was observed in the group of T2DM without IHD.

Upregulated miRNAs in the T2DM IHD group with the highest fold change and AUC value were miR-615-3p and miR-3147 (FC = 2.45 and 2.35; AUC = 0.877 and 0.858 respectively). A study by Zong et al. compared miRNA profiles in patients with acute myocardial infarction to controls. The results from RNA sequencing pointed out 96 up-, and 85 down-regulated miRNAs included miR-615-3p, which was also confirmed by qPCR validation. In this case, the AUC for miR-615-3p was 0.688 (47). MiR-3147,miR-1224-5p, miR-5196-3p, miR-6732-3p, and miR-548b-3p have never been previously described concerning T2DM or vascular disease. Their indication in our study may evidence that they are new potential biomarkers of IHD in T2DM. These results suggest that indicated miRNAs candidates have a high probability of being specific for the T2DM IHD phenotype and could play a pivotal role in the IHD diagnosis in T2DM patients.

The IPA analysis indicated 489 molecules regulated by identified miRNAs. Further analysis linked the target genes into a PPI network and extracted ten hub genes. Some of them are connected with cardiovascular abnormalities and T2DM. *C3aR1* showed elevated expression in obese patients compared to the control group (48). Research implicates that *CCR5* plays an important role in the initiation and progression of atherosclerosis (49). *CXCL6* and *CXCL8* belong to the cytokine family connected with heart failure and T2DM (50, 51). *ADORA1* gene encodes adenosine A1 receptor and, in the heart, it is expressed in cardiomyocytes, and its activation might promote angiogenesis (52). *ADORA1* is upregulated in the epicardial adipose tissue, which may be involved in IHD pathogenesis compared with mediastinal adipose tissue (53). Adenosine signalling has a crucial role in diabetes mellitus pathophysiology due to its modulation of insulin secretion and regulation of β-cell homeostasis (54). Our analysis showed that *APLNR*, which encodes apelin receptor, is a target gene for miR-5196-3p. Apelin showed vascular effects in numerous studies and under normal conditions, it lowers blood pressure (55, 56). Recent studies have found that apelin-mediated signalling is connected to heart failure and IHD. Furthermore, apelin participates in the pathology of diabetes by playing a pivotal role in increasing glucose uptake and insulin sensitivity (57). Moreover, a meta-analysis study showed that circulating apelin levels in humans are higher in T2DM patients than in healthy controls (58). In contrast, a study by Castan-Laurell et al. has proven that apelin can regulate blood glucose levels, and elevated levels of plasma apelin might be beneficial in reducing the risk of diabetes (59). Given that most of the presented miRNAs have not been previously described and are potentially associated with this gene involved in critical processes leading to IHD, special attention should be paid to it in further studies.

Ingenuity core analysis allowed us to identify which canonical pathways are dysregulated by tested upregulated miRNAs. Our results showed that one of the most important dysregulated canonical pathways in the T2DM IHD patients was endothelin-1 signalling. Endothelin-1 is a potent vasoconstrictor and pro-inflammatory protein and is an important contributor to the pathogenesis of hypertension, atherosclerosis, hypertrophy, and diabetes (60). Influenced by risk factors for cardiovascular disorders, its expression is altered, which plays an important role in the pathology of the cardiovascular system (61). Furthermore, patients with T2DM have increased vasoconstrictor activity induced by endothelin-1 (62). It has been proven that p38 MPAK has a crucial role in myocyte proliferation and apoptosis in the heart and participates in cardiac hypertrophy regulation (63). Interestingly Liang et al. conducted a study that confirms that miR-124 inhibits macrophage cells by targeting the p38/MAPK signalling pathway in the development of atherosclerosis. Mineralocorticoid biosynthesis is another canonical pathway that might be involved in IHD, especially at the atherosclerosis development (64, 65). Similarly, glucocorticoids and their receptors are also related to blood pressure regulation, atherosclerosis, and heart failure (66). Another important dysregulated canonical pathway is the pentose phosphate pathway which is a significant pathway for glucose metabolism, is required to synthesise ribonucleotides and produces a reduced form of nicotinamide adenine dinucleotide phosphate (NADPH), which is a key reductant in anabolic processes (67). This pathway participates in the T2DM pathogenesis (68). Melatonin signalling is another dysregulated canonical pathway in T2DM IHD patients. Melatonin has a considerable role in arterial blood pressure, insulin resistance, lipid and glucose metabolism, and control sleep-wake cycles. Patients with IHD have low melatonin production rates. Interestingly, this analysis also confirmed the importance of apelin signalling. Indeed, these identified canonical pathways play a significant role in the mechanisms leading to the development of cardiovascular diseases. It allows supposing that miRNAs determined by us play a relevant role in the regulation of genes associated with cardiovascular diseases and top dysregulated canonical pathways.

Nevertheless, we did not find any statistically significant correlation between the level of miRNAs and clinical parameters such as age, BMI, leucocytes, fasting glucose, HbA1c, diastolic blood pressure, cholesterol, triglycerides, or LDL level. However, those parameters are not specific only for IHD. Fibrinogen is not only an indicator of hypercoagulability but, as an acute-phase protein, is also an indicator of inflammation. In epidemiological and clinical studies, elevated blood fibrinogen levels are an independent risk factor for cardiovascular diseases (69–71). It has been proven that miRNAs can regulate fibrinogen production (72). Interestingly, we indicated a moderate positive correlation between levels of fibrinogen and miR-1224-5p, miR-3147, miR-5196-3p+miR-6732-3p, and miR-615-3p which have not been previously described as being related to this protein. We have indicated a moderate negative correlation between miR-548b-3p and platelets. The link between miRNAs and platelet in the development of IHD has been previously proven and summed up in a review by Stojkovic et al. (73). A strong or moderate positive correlation between all miRNA’s levels suggested they belong to the miRNA group, which dysregulation of mechanisms responsible for diabetic complications.

The area under the ROC curve remains a major criterion for diagnostic biomarkers. The ROC curve illustrates the relationship between diagnostic sensitivity and specificity and describes the diagnostic value of the tested parameters (74). To the best of our knowledge, there is no evidence in the literature describing the usefulness of miR-615-3p, miR-3147, (miR-1224-5p, miR-5196-3p+miR-6732-3p, miR-548b-3p) as a diagnostic tool for IHD in T2DM. The highest AUC values were observed in this study for miR-615-3p and miR-3147. Furthermore, other tested miRNA (miR-1224-5p, miR-5196-3p+miR-6732-3p, and miR-548b-3p) also showed high AUC results. The combined analysis showed that a logistic regression model consisting of miR-3147 and miR-615-3p demonstrates higher diagnostic accuracy than those miRNAs individually. These findings suggest that it would be beneficial to introduce such a panel in predicting IHD development in T2DM patients. High AUC scores for these miRNAs provide the groundwork for future confirmatory studies with a comprehensive validation in a larger cohort of patients. Additionally, AUC values for MIF and CXCL12, which play an important role in IHD, were below 0.500, disqualifying these two proteins as a good diagnostic tool for IHD diabetic patients.

MIF and CXCL12 are considered as a potential biomarker for heart diseases in patients with T2DM (75). However, one must consider that diabetes is also an inflammation state, and non-specific inflammatory parameters like MIF or CXCL12 can be elevated in patients with IHD or without it. Low AUC values for those proteins support our observations that the changes in miRNA levels could be better prognostic IHD biomarkers than the level of MIF or CXCL12 in T2DM patients.

Currently, the RT-qPCR technique, which we have used to validate results, remains the most commonly used approach for miRNA expression profiling due to its price, sensitivity, and specificity. The RT-qPCR analysis showed significantly increased expressions of miR-615-3p and miR-3147 in the serum of patients with T2DM who developed IHD, which confirms the reliability of the nCounter platform.

Presented data indicate a specific serum miRNA profile of patients with T2DM and IHD, and the different levels of selective miRNA expression might have a crucial role in further IHD diagnosis. We believe that identified circulating miRNAs might serve as new, non-invasive biomarkers for early detection of IHD in T2DM patients. However, this data should be considered preliminary. Additional studies on a larger cohort of patients are required to validate the predictive value of those miRNAs to prompt diagnosis of IHD in T2DM patients or better identification of at-risk individuals.

## Data Availability Statement

The original contributions presented in the study are publicly available. This data can be found below: https://www.ncbi.nlm.nih.gov/geo/query/acc.cgi?acc=GSE185845

## Ethics Statement

The studies involving human participants were reviewed and approved by The Bioethics Committee of the Medical University of Bialystok, Poland (approval numbers: R-I-002/583/2019 and APK.002.35.2021). The patients/participants provided their written informed consent to participate in this study.

## Author Contributions

Conceptualization, AB, MN, and AK; methodology, AB, MN, IS, ASz, and AK; formal analysis, AB, MN, IS, and WB, investigation, AB, ASz, JR, DO, KG, and MN; writing—original draft preparation, AB; writing—review and editing, AK, MN, and ASk; visualization, AB and MN; supervision, MN, SD, and AK. All authors have read and agreed to the published version of the manuscript.

## Funding

This study was funded by internal financing of the Medical University of Bialystok SUB/1/NN/21/001/4406 and was supported by the funds of the Ministry of Education and Science within the project “Excellence Initiative - Research University”.

## Conflict of Interest

The authors declare that the research was conducted in the absence of any commercial or financial relationships that could be construed as a potential conflict of interest.

## Publisher’s Note

All claims expressed in this article are solely those of the authors and do not necessarily represent those of their affiliated organizations, or those of the publisher, the editors and the reviewers. Any product that may be evaluated in this article, or claim that may be made by its manufacturer, is not guaranteed or endorsed by the publisher.
