# Supplementary material for: Serum miRNA Profile in Diabetic Patients With Ischemic Heart Disease as a Promising Non-Invasive Biomarker
Source: Front Endocrinol (Lausanne). 2022 May 18;13:888948. doi: 10.3389/fendo.2022.888948 (PMC9157821; doi:10.3389/fendo.2022.888948)
Supplement: Supplementary file 1 [file DataSheet_1.docx]

Supplementary Material

Serum miRNA profile in diabetic patients with ischemic heart disease as a promising non-invasive biomarker

Supplementary information content list:

**Supplementary Table 1**. Significantly dysregulated miRNAs between T2DM IHD and T2DM group.

**Supplementary Table 2**. Significantly dysregulated miRNAs between T2DM IHD and control group.

**Supplementary Table 3.** Significantly dysregulated miRNAs between T2DM and control group.

**Supplementary Table 4.** Significantly dysregulated miRNAs between T2DM IHD and IHD group.

**Supplementary Table 5**. Tested miRNAs and targeted genes.

**Supplementary Table 6.** Top 36 ingenuity canonical pathways from targets of differentially expressed miRNAs.

**Supplementary Table 7**. The levels of MIF and CXCL12 in serum from T2DM IHD, T2DM, and control patients.

**Supplementary Table 1.** Significantly dysregulated miRNAs between T2DM IHD and T2DM group.

| **T2DM IHD vs. T2DM** | | |
| --- | --- | --- |
| **miRNA** | **FC** | **FDR** |
| miR-498 | 3,12 | 0 |
| miR-3147 | 2,35 | 0 |
| miR-615-3p | 2,45 | 0 |
| miR-1303 | 2 | 0 |
| miR-1224-5p | 1,68 | 0 |
| miR-548d-3p | 1,78 | 0 |
| miR-548b-3p | 1,55 | 0,01 |
| miR-504-3p | 1,71 | 0,01 |
| miR-5196-3p+miR-6732-3p | 1,56 | 0,01 |
| miR-651-5p | 1,54 | 0,01 |
| miR-1266-5p | 1,64 | 0,01 |
| miR-4455 | 1,63 | 0,02 |
| miR-548o-3p+miR-548ah-3p+miR-548av-3p | 1,52 | 0,05 |
| miR-93-5p | 1,63 | 0,05 |

**Supplementary Table 2.** Significantly dysregulated miRNAs between T2DM IHD and control group.

| **T2DM IHD vs. control group** | | | | | | | | | | | | | | | | | |
| --- | --- | --- | --- | --- | --- | --- | --- | --- | --- | --- | --- | --- | --- | --- | --- | --- | --- |
| **miRNA** | **FC** | **FDR** | **miRNA** | **FC** | **FDR** | **miRNA** | **FC** | **FDR** | **miRNA** | **FC** | **FDR** | **miRNA** | **FC** | **FDR** | **miRNA** | **FC** | **FDR** |
| miR-497-5p | -13,94 | 0 | miR-539-5p | 1,62 | 0 | miR-508-5p | 1,87 | 0 | miR-4454+miR-7975 | 2,29 | 0 | miR-619-3p | 3,01 | 0 | miR-515-5p | 4,47 | 0 |
| miR-450a-2-3p | -10,04 | 0 | miR-105-5p | 1,63 | 0 | miR-892b | 1,88 | 0 | miR-99b-5p | 2,31 | 0 | miR-93-5p | 3,09 | 0 | miR-940 | 4,52 | 0 |
| miR-1272 | -9,95 | 0 | miR-624-3p | 1,64 | 0 | miR-3161 | 1,89 | 0 | miR-651-3p | 2,35 | 0 | miR-492 | 3,15 | 0 | miR-493-3p | 4,61 | 0 |
| miR-612 | -8,23 | 0 | miR-378h | 1,65 | 0 | miR-320b | 1,89 | 0 | miR-551a | 2,35 | 0 | miR-3150b-3p | 3,16 | 0 | miR-1276 | 4,61 | 0 |
| let-7i-5p | -7,7 | 0 | miR-374c-5p | 1,66 | 0 | miR-202-3p | 1,89 | 0 | miR-548ah-5p | 2,35 | 0 | miR-615-3p | 3,18 | 0 | miR-1244 | 7,34 | 0 |
| miR-199b-5p | -6,16 | 0 | miR-1270 | 1,67 | 0 | miR-200c-3p | 1,89 | 0 | miR-4448 | 2,37 | 0 | miR-500a-5p+miR-501-5p | 3,21 | 0 | miR-4455 | 8,28 | 0 |
| miR-26a-5p | -5,87 | 0 | miR-192-5p | 1,67 | 0 | miR-548j-3p | 1,91 | 0 | miR-1269b | 2,38 | 0 | miR-3202 | 3,26 | 0 | miR-6511a-5p | 8,88 | 0 |
| miR-210-3p | -5,49 | 0 | miR-206 | 1,71 | 0 | miR-548e-3p | 1,93 | 0 | miR-374b-5p | 2,41 | 0 | miR-25-3p | 3,26 | 0 | miR-194-5p | 9,56 | 0 |
| miR-937-3p | -4,36 | 0 | miR-219a-1-3p | 1,72 | 0 | miR-195-5p | 1,97 | 0 | miR-24-3p | 2,44 | 0 | miR-409-3p | 3,3 | 0 | miR-874-3p | 10,18 | 0 |
| miR-519c-3p | -3,8 | 0 | miR-323b-3p | 1,73 | 0 | miR-610 | 1,98 | 0 | miR-363-5p | 2,56 | 0 | miR-203a-3p | 3,33 | 0 | miR-134-5p+miR-6728-5p | 10,58 | 0 |
| miR-181a-3p | -3,41 | 0 | miR-1255b-5p | 1,74 | 0 | miR-1180-3p | 2,01 | 0 | miR-520e | 2,58 | 0 | miR-1266-5p | 3,33 | 0 | miR-155-5p | 10,82 | 0 |
| miR-370-3p | -3,1 | 0 | miR-30d-5p | 1,75 | 0 | miR-519e-3p | 2,02 | 0 | miR-1252-5p | 2,61 | 0 | miR-3192-5p | 3,34 | 0 | miR-302f | 10,85 | 0 |
| miR-34a-5p | -2,82 | 0 | miR-1233-3p | 1,77 | 0 | miR-3916 | 2,07 | 0 | miR-513c-5p | 2,61 | 0 | miR-1469 | 3,48 | 0 | miR-4431 | 11,2 | 0 |
| miR-107 | -2,74 | 0 | miR-30a-5p | 1,77 | 0 | miR-4532 | 2,08 | 0 | miR-4286 | 2,61 | 0 | miR-562 | 3,54 | 0 | miR-761 | 12,01 | 0 |
| miR-4707-5p | -2,74 | 0 | miR-98-3p | 1,77 | 0 | miR-614 | 2,08 | 0 | miR-874-5p | 2,75 | 0 | miR-125a-5p | 3,67 | 0 | miR-2117 | 15,65 | 0 |
| miR-1290 | -2,72 | 0 | miR-887-3p | 1,78 | 0 | miR-92a-3p | 2,09 | 0 | miR-651-5p | 2,78 | 0 | miR-361-5p | 3,67 | 0 | miR-218-5p | 18,5 | 0 |
| miR-1271-5p | 1,52 | 0 | miR-518b | 1,8 | 0 | miR-617 | 2,12 | 0 | miR-3147 | 2,79 | 0 | miR-548d-3p | 3,77 | 0 | miR-128-2-5p | 22,24 | 0 |
| miR-1224-5p | 1,52 | 0 | miR-548q | 1,81 | 0 | miR-98-5p | 2,19 | 0 | miR-1827 | 2,82 | 0 | miR-31-5p | 3,8 | 0 | miR-451a | -4,96 | 0,01 |
| miR-342-5p | 1,53 | 0 | miR-3127-5p | 1,81 | 0 | miR-671-3p | 2,23 | 0 | miR-296-5p | 2,87 | 0 | miR-328-3p | 3,95 | 0 | miR-2110 | -2,25 | 0,02 |
| miR-595 | 1,54 | 0 | miR-4521 | 1,83 | 0 | miR-29b-3p | 2,24 | 0 | miR-1908-3p | 2,91 | 0 | miR-1303 | 4,01 | 0 | miR-1285-5p | -2,19 | 0,02 |
| miR-3605-3p | 1,55 | 0 | miR-629-5p | 1,83 | 0 | miR-3136-5p | 2,28 | 0 | miR-615-5p | 2,96 | 0 | miR-498 | 4,03 | 0 | miR-1305 | -2,17 | 0,02 |
| let-7d-5p | 1,58 | 0 | miR-1288-3p | 1,84 | 0 | miR-3164 | 2,29 | 0 | miR-182-3p | 2,99 | 0 | miR-181c-5p | 4,14 | 0 | miR-5196-3p+miR-6732-3p | 1,53 | 0,02 |
| miR-548b-3p | 1,59 | 0 | miR-1304-3p | 30,99 | 0 | miR-151a-3p | 24,65 | 0 | miR-300 | -2,56 | 0,01 | let-7b-5p | 56,67 | 0 | miR-548y | -2,09 | 0,04 |
| miR-4458 | 1,6 | 0 | miR-890 | 44,83 | 0 | miR-15a-5p | 26,54 | 0 | miR-25-5p | -2,31 | 0,01 | miR-504-3p | 189,43 | 0 | miR-764 | -1,99 | 0,04 |
| miR-146b-3p | 1,6 | 0 | miR-4435 | 48,57 | 0 | let-7g-5p | 29,6 | 0 | miR-607 | -2,34 | 0,02 | miR-544a | 4,3 | 0 | miR-873-3p | 1,73 | 0,04 |
| miR-181b-5p+miR-181d-5p | 1,61 | 0 | miR-758-3p+miR-411-3p | 1,9 | 0 | miR-519b-5p+miR-519c-5p+miR-523-5p+miR-518e-5p+miR-522-5p+miR-519a-5p | 2,08 | 0 | miR-548o-3p+miR-548ah-3p+miR-548av-3p | 2,28 | 0 | miR-548ak | 4,33 | 0 |  |  |  |

**Supplementary Table 3.** Significantly dysregulated miRNAs between T2DM and control group.

| **T2DM vs. control group** | | | | | | | | | | | | | | | | | |  |
| --- | --- | --- | --- | --- | --- | --- | --- | --- | --- | --- | --- | --- | --- | --- | --- | --- | --- | --- |
| **miRNA** | **FC** | **FDR** | **miRNA** | **FC** | **FDR** | **miRNA** | **FC** | **FDR** | **miRNA** | **FC** | **FDR** | **miRNA** | **FC** | **FDR** | **miRNA** | **FC** | **FDR** | |
| miR-497-5p | -15,35 | 0 | miR-30d-5p | 1,71 | 0 | miR-617 | 2,12 | 0 | miR-363-5p | 2,49 | 0 | miR-125a-5p | 3,95 | 0 | miR-105-5p | 1,7 | 0 | |
| miR-450a-2-3p | -12,21 | 0 | miR-323b-3p | 1,71 | 0 | miR-1266-5p | 2,16 | 0 | miR-182-3p | 2,49 | 0 | miR-361-5p | 4,13 | 0 | miR-128-2-5p | 24,02 | 0 | |
| miR-1272 | -10,96 | 0 | miR-3605-3p | 1,72 | 0 | miR-1269b | 2,17 | 0 | miR-1180-3p | 2,53 | 0 | miR-548ak | 4,28 | 0 | miR-15a-5p | 27,98 | 0 | |
| miR-612 | -9,3 | 0 | miR-3161 | 1,73 | 0 | miR-320b | 2,17 | 0 | miR-3164 | 2,54 | 0 | miR-181c-5p | 4,3 | 0 | let-7g-5p | 30,6 | 0 | |
| let-7i-5p | -8,37 | 0 | miR-1288-3p | 1,73 | 0 | miR-3916 | 2,18 | 0 | miR-4448 | 2,61 | 0 | miR-25-3p | 4,43 | 0 | miR-1304-3p | 37,75 | 0 | |
| miR-199b-5p | -6,89 | 0 | miR-206 | 1,74 | 0 | miR-1233-3p | 2,18 | 0 | miR-874-5p | 2,75 | 0 | miR-515-5p | 4,68 | 0 | miR-890 | 51,89 | 0 | |
| miR-26a-5p | -6,37 | 0 | miR-892b | 1,75 | 0 | miR-202-3p | 2,21 | 0 | miR-1827 | 2,81 | 0 | miR-328-3p | 4,69 | 0 | miR-4435 | 60,02 | 0 | |
| miR-210-3p | -5,62 | 0 | miR-374c-5p | 1,75 | 0 | miR-29b-3p | 2,21 | 0 | miR-520e | 2,83 | 0 | miR-940 | 4,76 | 0 | let-7b-5p | 61,16 | 0 | |
| miR-937-3p | -4,17 | 0 | miR-376a-2-5p | 1,78 | 0 | miR-1303 | 2,22 | 0 | miR-1252-5p | 2,93 | 0 | miR-31-5p | 4,88 | 0 | miR-504-3p | 120,65 | 0 | |
| miR-1290 | -3,65 | 0 | miR-4532 | 1,81 | 0 | miR-374b-5p | 2,26 | 0 | miR-513c-5p | 2,98 | 0 | miR-544a | 4,92 | 0 | miR-300 | -2,61 | 0,01 | |
| miR-519c-3p | -3,61 | 0 | miR-219a-1-3p | 1,81 | 0 | miR-192-5p | 2,26 | 0 | miR-492 | 3,15 | 0 | miR-493-3p | 4,93 | 0 | miR-301a-5p | -2,52 | 0,01 | |
| miR-4707-5p | -3,54 | 0 | miR-518b | 1,84 | 0 | miR-548d-3p | 2,27 | 0 | miR-619-3p | 3,2 | 0 | miR-1276 | 5,01 | 0 | miR-2110 | -2,45 | 0,01 | |
| miR-181a-3p | -3,53 | 0 | miR-651-5p | 1,85 | 0 | miR-595 | 2,28 | 0 | miR-3192-5p | 3,24 | 0 | miR-4455 | 5,37 | 0 | miR-543 | -2,38 | 0,01 | |
| miR-370-3p | -3,41 | 0 | let-7d-5p | 1,88 | 0 | miR-671-3p | 2,32 | 0 | miR-1908-3p | 3,25 | 0 | miR-1244 | 7,64 | 0 | miR-548e-5p | -2,34 | 0,01 | |
| miR-107 | -3,29 | 0 | miR-610 | 1,88 | 0 | miR-92a-3p | 2,33 | 0 | miR-3150b-3p | 3,32 | 0 | miR-302f | 8,28 | 0 | miR-498 | 1,54 | 0,01 | |
| miR-34a-5p | -3,09 | 0 | miR-4521 | 1,9 | 0 | miR-508-5p | 2,33 | 0 | miR-615-5p | 3,37 | 0 | miR-6511a-5p | 9,76 | 0 | miR-606 | 1,56 | 0,01 | |
| miR-25-5p | -2,63 | 0 | miR-548e-3p | 1,9 | 0 | miR-887-3p | 2,33 | 0 | miR-296-5p | 3,43 | 0 | miR-194-5p | 10,66 | 0 | miR-548ah-5p | 2,25 | 0,01 | |
| miR-767-3p | 1,52 | 0 | miR-1255b-5p | 1,94 | 0 | miR-98-5p | 2,4 | 0 | miR-203a-3p | 3,46 | 0 | miR-874-3p | 11,75 | 0 | miR-607 | -2,37 | 0,02 | |
| miR-944 | 1,53 | 0 | miR-98-3p | 1,94 | 0 | miR-651-3p | 2,41 | 0 | miR-562 | 3,62 | 0 | miR-4431 | 12,32 | 0 | miR-1295a | -2,23 | 0,02 | |
| miR-539-5p | 1,54 | 0 | miR-1270 | 1,96 | 0 | miR-3136-5p | 2,42 | 0 | miR-500a-5p+miR-501-5p | 3,63 | 0 | miR-761 | 12,97 | 0 | miR-1285-5p | -2,19 | 0,02 | |
| miR-524-3p | 1,55 | 0 | miR-195-5p | 1,97 | 0 | miR-614 | 2,44 | 0 | miR-3202 | 3,65 | 0 | miR-155-5p | 13,65 | 0 | miR-1305 | -2,16 | 0,02 | |
| miR-624-3p | 1,6 | 0 | miR-4458 | 1,99 | 0 | miR-99b-5p | 2,46 | 0 | miR-1469 | 3,82 | 0 | miR-151a-3p | 18,96 | 0 | miR-133a-3p | -2,07 | 0,02 | |
| miR-30a-5p | 1,62 | 0 | miR-93-5p | 2 | 0 | miR-24-3p | 2,47 | 0 | miR-4286 | 3,82 | 0 | miR-2117 | 22,84 | 0 | miR-95-3p | -2,2 | 0,03 | |
| miR-655-3p | 1,63 | 0 | miR-519e-3p | 2,01 | 0 | miR-551a | 2,49 | 0 | miR-409-3p | 3,87 | 0 | miR-218-5p | 23,8 | 0 | miR-186-5p | -2,2 | 0,03 | |
| miR-1291 | 1,64 | 0 | miR-629-5p | 2,06 | 0 | miR-519b-5p+miR-519c-5p+miR-523-5p+miR-518e-5p+miR-522-5p+miR-519a-5p | 1,96 | 0 | miR-181b-5p+miR-181d-5p | 2,05 | 0 | miR-758-3p+miR-411-3p | 3,48 | 0 | miR-548y | -2,09 | 0,05 | |
| miR-342-5p | 1,67 | 0 | miR-200c-3p | 2,06 | 0 | miR-548o-3p+miR-548ah-3p+miR-548av-3p | 1,58 | 0 | miR-4454+miR-7975 | 2,72 | 0 | miR-134-5p+miR-6728-5p | 11,74 | 0 |  |  |  | |

**Supplementary Table 4.** Significantly dysregulated miRNAs between T2DM IHD and IHD group.

| **T2DM IHD vs. IHD** | | | | | | | | | | | | | | | | | |  |
| --- | --- | --- | --- | --- | --- | --- | --- | --- | --- | --- | --- | --- | --- | --- | --- | --- | --- | --- |
| **miRNA** | **FC** | **FDR** | **miRNA** | **FC** | **FDR** | **miRNA** | **FC** | **FDR** | **miRNA** | **FC** | **FDR** | **miRNA** | **FC** | **FDR** | **miRNA** | **FC** | **FDR** | |
| miR-4435 | 58,26 | 0 | miR-3136-5p | 3,03 | 0 | miR-548a-5p | -1,88 | 0 | miR-10b-5p | -2,5 | 0 | miR-34c-3p | -18,51 | 0 | miR-28-3p | -2,83 | 0,01 | |
| miR-890 | 56,33 | 0 | miR-24-3p | 2,91 | 0 | miR-107 | -1,89 | 0 | miR-1295a | -2,6 | 0 | miR-497-5p | -75,54 | 0 | miR-937-3p | -4,08 | 0,01 | |
| let-7b-5p | 52,3 | 0 | miR-548ah-5p | 2,87 | 0 | miR-208b-3p | -1,89 | 0 | miR-134-3p | -2,62 | 0 | miR-21-5p | -528,88 | 0 | miR-409-3p | 3,75 | 0,02 | |
| miR-1304-3p | 40,82 | 0 | miR-551a | 2,86 | 0 | miR-378f | -1,89 | 0 | miR-543 | -2,62 | 0 | miR-504-3p | 256,21 | 0,01 | miR-513c-5p | 3,41 | 0,02 | |
| miR-15a-5p | 33,69 | 0 | miR-324-3p | 2,65 | 0 | miR-3614-5p | -1,93 | 0 | miR-25-5p | -2,67 | 0 | miR-302f | 13,26 | 0,01 | miR-617 | 2,73 | 0,02 | |
| let-7g-5p | 27,85 | 0 | miR-892b | 2,63 | 0 | miR-933 | -1,97 | 0 | miR-186-5p | -2,75 | 0 | miR-1244 | 8,78 | 0,01 | miR-548e-3p | 2,55 | 0,02 | |
| miR-128-2-5p | 27,74 | 0 | miR-887-3p | 2,61 | 0 | miR-4461 | -1,98 | 0 | miR-607 | -2,77 | 0 | miR-4455 | 6,1 | 0,01 | miR-195-5p | 2,53 | 0,02 | |
| miR-218-5p | 25,03 | 0 | miR-4532 | 2,59 | 0 | miR-369-3p | -1,99 | 0 | miR-1255a | -2,79 | 0 | miR-940 | 5,74 | 0,01 | miR-98-3p | 2,41 | 0,02 | |
| miR-2117 | 18,4 | 0 | miR-182-3p | 2,57 | 0 | miR-1323 | -2 | 0 | miR-888-5p | -2,81 | 0 | miR-31-5p | 5,43 | 0,01 | miR-374c-5p | 2,33 | 0,02 | |
| miR-761 | 15,04 | 0 | miR-4458 | 2,52 | 0 | miR-1262 | -2,01 | 0 | miR-1305 | -2,9 | 0 | miR-125a-5p | 4,79 | 0,01 | miR-767-3p | 2,19 | 0,02 | |
| miR-4431 | 12,32 | 0 | miR-3916 | 2,46 | 0 | miR-549a | -2,01 | 0 | miR-1296-3p | -2,97 | 0 | miR-361-5p | 4,3 | 0,01 | miR-624-3p | 1,88 | 0,02 | |
| miR-874-3p | 12 | 0 | miR-610 | 2,36 | 0 | miR-1228-3p | -2,05 | 0 | miR-590-5p | -2,97 | 0 | miR-363-5p | 3,5 | 0,01 | miR-297 | 1,87 | 0,02 | |
| miR-194-5p | 11,18 | 0 | miR-30d-5p | 2,33 | 0 | miR-1268b | -2,07 | 0 | miR-1973 | -3,05 | 0 | miR-1908-3p | 3,38 | 0,01 | miR-9-5p | 1,74 | 0,02 | |
| miR-6511a-5p | 10,37 | 0 | miR-518b | 2,21 | 0 | miR-23a-3p | -2,08 | 0 | miR-183-5p | -3,07 | 0 | miR-320b | 3,01 | 0,01 | miR-376c-3p | 1,54 | 0,02 | |
| miR-493-3p | 5,92 | 0 | miR-219a-1-3p | 2,02 | 0 | miR-1197 | -2,09 | 0 | miR-504-5p | -3,21 | 0 | miR-651-3p | 2,88 | 0,01 | miR-924 | 1,53 | 0,02 | |
| miR-1276 | 5,8 | 0 | miR-587 | -1,57 | 0 | miR-597-5p | -2,1 | 0 | miR-30e-5p | -3,25 | 0 | miR-374b-5p | 2,85 | 0,01 | miR-455-5p | -1,52 | 0,02 | |
| miR-515-5p | 5,74 | 0 | miR-301a-3p | -1,63 | 0 | miR-199a-5p | -2,12 | 0 | miR-370-3p | -3,25 | 0 | miR-4448 | 2,85 | 0,01 | miR-626 | -1,53 | 0,02 | |
| miR-548ak | 5,66 | 0 | miR-5010-3p | -1,63 | 0 | miR-30a-3p | -2,13 | 0 | miR-548y | -3,29 | 0 | miR-4521 | 2,82 | 0,01 | miR-210-5p | -1,56 | 0,02 | |
| miR-181c-5p | 5,27 | 0 | miR-922 | -1,66 | 0 | miR-33b-5p | -2,14 | 0 | miR-1285-5p | -3,35 | 0 | miR-519e-3p | 2,65 | 0,01 | miR-454-3p | -1,56 | 0,02 | |
| miR-544a | 5,16 | 0 | miR-767-5p | -1,67 | 0 | miR-584-3p | -2,14 | 0 | miR-301a-5p | -3,41 | 0 | miR-3164 | 2,64 | 0,01 | miR-513a-3p | -1,71 | 0,02 | |
| miR-3192-5p | 4,63 | 0 | miR-200a-3p | -1,68 | 0 | miR-184 | -2,2 | 0 | miR-519c-3p | -3,43 | 0 | miR-4286 | 2,6 | 0,01 | miR-451a | -7,4 | 0,02 | |
| miR-328-3p | 4,59 | 0 | miR-4488 | -1,7 | 0 | miR-764 | -2,2 | 0 | miR-300 | -3,51 | 0 | miR-30a-5p | 2,59 | 0,01 | miR-1252-5p | 3,17 | 0,03 | |
| miR-3202 | 4,57 | 0 | miR-495-5p | -1,7 | 0 | miR-335-5p | -2,21 | 0 | miR-181a-3p | -3,52 | 0 | miR-105-5p | 2,38 | 0,01 | miR-1233-3p | 2,45 | 0,03 | |
| miR-296-5p | 4,39 | 0 | miR-211-5p | -1,71 | 0 | miR-525-5p | -2,21 | 0 | miR-585-3p | -3,6 | 0 | miR-520b | 1,99 | 0,01 | miR-192-5p | 2,06 | 0,03 | |
| miR-562 | 4,35 | 0 | miR-887-5p | -1,74 | 0 | miR-10a-5p | -2,22 | 0 | miR-644a | -4,07 | 0 | miR-342-5p | 1,95 | 0,01 | miR-152-3p | 1,98 | 0,03 | |
| miR-492 | 4,23 | 0 | miR-487a-3p | -1,75 | 0 | miR-639 | -2,24 | 0 | miR-612 | -4,6 | 0 | miR-548h-5p | 1,84 | 0,01 | miR-221-5p | 1,8 | 0,03 | |
| miR-619-3p | 4,09 | 0 | miR-548m | -1,75 | 0 | miR-548ar-3p | -2,26 | 0 | miR-33a-5p | -4,78 | 0 | miR-495-3p | 1,7 | 0,01 | miR-27a-3p | -1,54 | 0,03 | |
| miR-1469 | 4,03 | 0 | miR-337-5p | -1,77 | 0 | miR-939-5p | -2,28 | 0 | miR-210-3p | -5,74 | 0 | miR-877-5p | -1,53 | 0,01 | miR-548g-3p | -1,79 | 0,03 | |
| miR-520e | 3,99 | 0 | miR-133a-3p | -1,78 | 0 | miR-4536-5p | -2,31 | 0 | miR-758-5p | -5,97 | 0 | miR-891a-5p | -1,59 | 0,01 | miR-2110 | -2,63 | 0,03 | |
| miR-3150b-3p | 3,88 | 0 | miR-26b-5p | -1,79 | 0 | miR-514a-3p | -2,32 | 0 | miR-3130-3p | -6,26 | 0 | miR-591 | -1,61 | 0,01 | miR-151a-3p | 8,14 | 0,04 | |
| miR-1827 | 3,82 | 0 | miR-203a-5p | -1,82 | 0 | miR-1200 | -2,33 | 0 | miR-26a-5p | -6,32 | 0 | miR-512-5p | -1,73 | 0,01 | miR-1180-3p | 2,85 | 0,04 | |
| miR-203a-3p | 3,75 | 0 | miR-378i | -1,82 | 0 | miR-542-3p | -2,33 | 0 | miR-199b-5p | -7,1 | 0 | miR-643 | -1,75 | 0,01 | miR-342-3p | 1,8 | 0,04 | |
| miR-25-3p | 3,61 | 0 | miR-301b-5p | -1,84 | 0 | miR-378g | -2,34 | 0 | miR-450a-2-3p | -11,01 | 0 | miR-199a-3p+miR-199b-3p | -1,77 | 0,01 | miR-4454+miR-7975 | 1,7 | 0,04 | |
| miR-615-5p | 3,6 | 0 | miR-582-5p | -1,86 | 0 | miR-328-5p | -2,4 | 0 | miR-1272 | -11,92 | 0 | miR-362-5p | -1,86 | 0,01 | miR-34a-5p | -2,97 | 0,04 | |
| miR-1269b | 3,54 | 0 | miR-4536-3p | -1,87 | 0 | miR-500a-5p+miR-501-5p | 4,59 | 0 | miR-758-3p+miR-411-3p | 2,62 | 0 | miR-95-3p | -2,15 | 0,01 | miR-526a+miR-518c-5p+miR-518d-5p | -1,67 | 0,04 | |
| miR-874-5p | 3,3 | 0 | miR-134-5p+miR-6728-5p | 12,62 | 0 | miR-579-5p | -2,41 | 0 | let-7i-5p | -16,56 | 0 | miR-519b-5p+miR-519c-5p+miR-523-5p+miR-518e-5p+miR-522-5p+miR-519a-5p | 2,27 | 0,01 | miR-548o-3p+miR-548ah-3p+miR-548av-3p | 3,66 | 0,04 | |
| miR-98-5p | 3,46 | 0 | miR-4516 | -1,88 | 0 | miR-126-3p | -2,48 | 0 | miR-181b-5p+miR-181d-5p | 1,86 | 0 | miR-365a-3p+miR-365b-3p | -2,45 | 0,1 |  |  |  | |

**Supplementary Table 5**. Tested miRNAs and targeted genes.

| **miRNA** | **targetet genes** | **number of targets** |
| --- | --- | --- |
| miR-1224-5p | ABCD1, ACTL6B, ADORA1, AMY2A, ARHGEF19, ATP4A, BEND4, C14orf119, C3orf56, CBX3, CD160, CLEC4F, CLNS1A, COPB1, CPNE1, CTXN2, CXCL6, DNAAF3, EFCAB10, EIF4E1B, ERH, GJB5, GMFG, GPSM3, GPX5, KRT72, KRTAP8-1, KRTAP9-6, KYAT1, LBP, LCE3D, LCE3E, MADD, MAP7D3, MED20, MS4A6E, MYBPHL, NAT14, NPHS2, PCMT1, PLCB3, PMAIP1, PPP1R27, PRRX1, PSME3, RHEX, RNASE2, RNASE3, S100A12, SCML4, SDF2L1, SIAH3, SIRT6, TMEM213, TMEM221, TNNC1, TROAP, TUBB2A, UCHL1, ZNF100, ZNF107, ZNF117, ZNF141, ZNF195, ZNF208, ZNF257, ZNF430, ZNF676, ZNF728, ZNF99 | 70 |
| miR-3147 | ANKDD1A, ANKRD54, AP3S2, APRT, ATP5IF1, BAIAP2L2, C10orf126, C1QB, C22orf24, CCDC120, CCDC39, CD53, CLDN6, CLEC4G, CNOT2, COMMD7, CREB3L1, CTIF, DNASE1, DNASE1L2, EFCC1, EGFL7, F2RL2, FAM217A, FOXC2, FXR2, FZD2, G6PD, GABRA1, GALNT12, GDF1, GIPC3, GNAI2, GSDMA, H1-4, HSD3B1, HSD3B2, ICOSLG, KCND1, LAPTM5, LCE3E, LTBP1, LYRM9, MAN1C1, MFSD6L, MS4A14, OPRL1, PAFAH2, PGPEP1L, PLA2G2D, PLEKHS1, PLLP, PPHLN1, PPIL3, PPP5D1, PRF1, PSMB11, PTPN2, RNF4, SLC19A1, SLC22A9, SLC38A5, SLC39A8, SLC4A1, SNRNP25, SPOUT1, STX8, TGFA, THEM6, TMEM129, TMEM169, TMEM231, TMEM92, TMEM97, TNR, TPD52L3, UBD, ULBP1, VPS52, ZBED3, ZBTB8A, ZNF212, ZNF584, ZNF687, ZNF83 | 85 |
| miR-5196-3p | ABHD11, AFAP1L1, APLNR, ARMC1, ATP1A4, ATP5IF1, ATXN7L3B, BEST2, BHMT2, BRMS1L, C12orf60, C19orf25, C3AR1, C9orf47, CCR5, CDC42EP4, CDK4, CFAP221, CISH, CLPP, CMKLR1, COX7C, CTSD, CYP3A5, DST, EEF1AKMT3, EID2, EREG, ERG, FAM169A, FAN1, FANCD2, FAS, GABRA6, GKN2, GLRX2, GNAS, GPIHBP1, GRAMD2A, GSX2, HELLS, HMGB1, HNRNPA2B1, HNRNPC, IFT88, IL12B, ISCA2, LAS1L, LIF, LMOD1, LRRC27, LRRC38, LRRC42, LSM8, LY6D, LYSMD1, MAP3K13, MGA, MMP20, MTRNR2L5, MYT1L, NCS1, NFE2, NIPAL4, NR2E1, NUDT10, NXPH3, OR10H2, P3H3, PDC, PDGFA, PDZD11, PEG10, PHF1, PLA2G3, PNMA5, POLR3GL, POMGNT1, PPP1R10, PRKACG, PSD2, PTMS, RHOB, RND1, RNF168, RORC, RSPH10B, SCGN, SMARCC2, SMCP, SPANXN1, SPANXN5, SSBP1, STK4, TBCA, TCL1A, TCTA, TFE3, TM7SF2, TMEM139, TMEM38A, TPD52L3, TRIM44, TRIM68, TRPV2, TTC39C, TUBB6, TVP23C-CDRT4, UNC13A, UNC93A, WDR18, YPEL4, ZDHHC5, ZNF502, ZNF584, ZSWIM3 | 116 |
| miR-548b-3p | ARL13A, ARPC2, ATP5MD, BECN1, C1orf185, C4orf3, CACHD1, CAP1, CELA1, CHORDC1, CIP2A, CLP1, COX7C, DLEU7, EXO5, FAM222B, FGL2, GABRG2, HAMP, HAO1, HMCES, IMP3, ISM1, JUNB, KRTAP9-3, LAGE3, LDB2, LMO7, MAP3K13, MTA2, NAA20, NAMPT, NPAS3, OGG1, OR2A4, OR2H1, OR4K2, PATE2, PCDHB13, PON2, PROK2, PTGS1, RPE, RPEL1, RPL37, RPS20, SDR39U1, SLC2A3, TFRC, TGFBRAP1, TMED4, TRAM1L1, TST, UBE2E2, WNT2, YWHAE | 56 |
| miR-615-3p | ABHD17A, APOB, AR, ASGR1, BLACE, BLOC1S1, C10orf67, C5orf38, CELF2, CLIP2, COL18A1, DAND5, DBX2, DNAH10OS, DUSP28, DUSP9, EHMT1, ENSA, EYA2, FAM219A, FBXO38, FCMR, GDPD2, GP5, GPR27, GRAPL, GSC2, HIRIP3, HOXC6, HSF1, HSPB7, IQCE, KCNK12, KIAA1614, KLF16, KRTAP5-11, LCOR, LRRC73, LTBP3, LTBP4, LYPD1, MAFA, MAP7D2, MAPK13, MAX, MEF2A, MEX3B, MICALL2, MOB3A, NELFCD, NOL3, NTF4, NUBP2, NUP62, PODXL, POLR3B, PRKCG, PRR5L, PRRT2, PRSS22, PRX, PSMG4, PTPN7, RAB31, RALBP1, RASL12, RAX, SEMA3G, SEMA4B, SLC26A10, SLC8A2, SMARCE1, SMDT1, SMIM20, SPIN1, ST14, SUPT16H, TEKT1, TFF3, THEM6, TLE2, TM9SF2, TOMM7, TRIM7, USP44, VSTM2L, ZBP1, ZFAND2A, ZNF205 | 89 |
| miR-6732-3p | ACAD11, ANKRD1, BAMBI, BLOC1S5, CASP6, CDKN2B, CFAP43, CHORDC1, CLASP2, CNN1, CNNM4, CRCP, CXCL8, DAND5, DCAF4L1, DDHD1, DOK5, EIF5A2, EXOC6, FIGLA, GPATCH2L, GRIN2B, HAVCR1, KLRG2, KRTAP22-2, LIG1, LSMEM1, MFSD13A, MPDU1, MRPL50, MTRNR2L1, MTRNR2L10, MTRNR2L11, MTRNR2L3, MTRNR2L4, MTRNR2L5, MTRNR2L7, MYO19, NCK2, NRBF2, OR56B4, OR9A2, PER2, PLD1, PLD6, PPP3R2, PSTPIP2, RPL23, RPP21, RPS15A, RSPO1, SDC1, SEC14L1, SF3A3, SH3BGR, SLC25A33, SOX3, SPP1, SSH2, STK32B, THOC6, TIFAB, TNFSF8, TNNI1, TRAPPC2, TRNT1, VPS50, WDR55, ZBTB37, ZNF487, ZNF492, ZNF705B, ZNF880 | 73 |

**Supplementary Table 6.** Top 36 ingenuity canonical pathways from targets of differentially expressed miRNAs.

|  | **Ingenuity Canonical Pathways** | **p-value** | **Molecules** | **number of molecues** |
| --- | --- | --- | --- | --- |
| 1 | Endothelin-1 Signaling | 0,003 | CASP6, GNAI2, GNAS, MAPK13, PLA2G2D, PLA2G3, PLCB3, PLD1, PLD6, PRKCG, PTGS1 | 11 |
| 2 | Airway Inflammation in Asthma | 0,006 | CXCL8, IL12B, RNASE2, RNASE3 | 4 |
| 3 | Antioxidant Action of Vitamin C | 0,010 | MAPK13, PLA2G2D, PLA2G3, PLCB3, PLD1, PLD6, SLC2A3 | 7 |
| 4 | Molecular Mechanisms of Cancer | 0,010 | CASP6, CDK4, CDKN2B, FANCD2, FAS, FZD2, GNAI2, GNAS, MAPK13, MAX, PLCB3, PMAIP1, PRKACG, PRKCG, RALBP1, RHOB, RND1, WNT2 | 18 |
| 5 | Phospholipases | 0,012 | PLA2G2D, PLA2G3, PLCB3, PLD1, PLD6 | 5 |
| 6 | Mineralocorticoid Biosynthesis | 0,013 | HSD3B1, HSD3B2 | 2 |
| 7 | Gαq Signaling | 0,014 | GNAI2, GNAS, PLCB3, PLD1, PLD6, PPP3R2, PRKCG, RHOB, RND1 | 9 |
| 8 | p38 MAPK Signaling | 0,017 | FAS, HSPB7, MAPK13, MAX, MEF2A, PLA2G2D, PLA2G3 | 7 |
| 9 | Glucocorticoid Biosynthesis | 0,017 | HSD3B1, HSD3B2 | 2 |
| 10 | Tumoricidal Function of Hepatic Natural Killer Cells | 0,017 | CASP6, FAS, PRF1 | 3 |
| 11 | Apelin Liver Signaling Pathway | 0,019 | APLNR, COL18A1, FAS | 3 |
| 12 | Pentose Phosphate Pathway | 0,021 | G6PD, RPE | 2 |
| 13 | Role of NFAT in Cardiac Hypertrophy | 0,021 | GNAI2, GNAS, LIF, MAPK13, MEF2A, PLCB3, PPP3R2, PRKACG, PRKCG, SLC8A2 | 9 |
| 14 | Melatonin Signaling | 0,022 | GNAI2, PLCB3, PRKACG, PRKCG, RORC | 5 |
| 15 | Axonal Guidance Signaling | 0,024 | ARPC2, FZD2, GNAI2, GNAS, MMP20, NCK2, NTF4, PDGFA, PLCB3, PPP3R2, PRKACG, PRKCG, RND1, SEMA3G, SEMA4B, TUBB2A, TUBB6, WNT2 | 18 |
| 16 | Apelin Cardiomyocyte Signaling Pathway | 0,024 | APLNR, GNAI2, MAPK13, PLCB3, PRKCG, SLC8A2 | 6 |
| 17 | CCR3 Signaling in Eosinophils | 0,025 | GNAI2, GNAS, MAPK13, PLA2G2D, PLA2G3, PLCB3, PRKCG | 7 |
| 18 | Protein Kinase A Signaling | 0,026 | DUSP9, EYA2, GNAI2, GNAS, H1-4, PALM2AKAP2, PLCB3, PLD6, PPP1R10, PPP3R2, PRKACG, PRKCG, PTPN2, PTPN7, YWHAE | 15 |
| 19 | Myc Mediated Apoptosis Signaling | 0,028 | FAS, MAX, PMAIP1, PRKACG | 4 |
| 20 | Sumoylation Pathway | 0,029 | AR, FAS, RHOB, RND1, RNF4, SLC19A1 | 6 |
| 21 | HMGB1 Signaling | 0,029 | CXCL8, HMGB1, IL12B, LIF, MAPK13, RHOB, RND1, TNFSF8 | 5 |
| 22 | Androgen Biosynthesis | 0,030 | HSD3B1, HSD3B2 | 2 |
| 23 | BER pathway | 0,030 | LIG1, OGG1 | 2 |
| 24 | Gap Junction Signaling | 0,032 | GNAI2, GNAS, PLCB3, PPP3R2, PRKACG, PRKCG, SMARCC2, TUBB2A, TUBB6 | 9 |
| 25 | cAMP-mediated signaling | 0,032 | ADORA1, APLNR, DUSP9, GNAI2, GNAS, OPRL1, PALM2AKAP2, PLD6, PPP3R2, PRKACG | 10 |
| 26 | Corticotropin Releasing Hormone Signaling | 0,039 | GNAI2, GNAS, MAPK13, MEF2A, PRKACG, PRKCG, SMARCC2 | 7 |
| 27 | Dopamine-DARPP32 Feedback in cAMP Signaling | 0,040 | GNAI2, GNAS, GRIN2B, PLCB3, PPP1R10, PPP3R2, PRKACG, PRKCG | 8 |
| 28 | Superpathway of Methionine Degradation | 0,040 | BHMT2, EEF1AKMT3, EHMT1 | 3 |
| 29 | Role of IL-17A in Psoriasis | 0,041 | CXCL6, CXCL8 | 2 |
| 30 | Choline Biosynthesis III | 0,041 | PLD1, PLD6 | 2 |
| 31 | Parkinson's Signaling | 0,041 | MAPK13, UCHL1 | 2 |
| 32 | Apelin Adipocyte Signaling Pathway | 0,042 | APLNR, GNAI2, GPX5, MAPK13, PRKACG | 5 |
| 33 | Methionine Salvage II (Mammalian) | 0,046 | BHMT2 | 1 |
| 34 | Thyroid Hormone Biosynthesis | 0,046 | CTSD | 1 |
| 35 | Neuroinflammation Signaling Pathway | 0,047 | CXCL8, FAS, GABRA1, GABRG2, GRIN2B, HMGB1, IL12B, MAPK13, PLA2G2D, PLA2G3, PPP3R2, PRKCG | 12 |
| 36 | Sphingosine-1-phosphate Signaling | 0,048 | CASP6, GNAI2, PDGFA, PLCB3, RHOB, RND1 | 6 |

Supplementary Table 7. The levels of MIF and CXCL12 in serum from T2DM IHD, T2DM, and control patients. Median and range results are shown. Significance: ANOVA Kruskal-Wallis comparison test; **** p<0.0001 in comparison to control group T2DM (-) IHD (-). a Significantly different from the control group.

|  | **control group T2DM (-) IHD (-) (*n* = 16)** | **T2DM (*n* = 20)** | **T2DM IHD (*n* = 24)** |
| --- | --- | --- | --- |
| **MIF ng/ml** | 0.66 (0.20 - 1.94) | 0.85 (0.17 - 6.60) | 0.65 (0.23 - 2.38) |
| **CXCL12 pg/ml** | 146.13 (47.38 - 183.14) | 2158.37 (508.38 - 2598.33)^a****^ | 2001.54 (807.11 - 3024.30)^a^**** |
